# Supplementary material for: Mining Indonesian Microbial Biodiversity for Novel Natural Compounds by a Combined Genome Mining and Molecular Networking Approach
Source: Mar Drugs. 2021 May 28;19(6):316. doi: 10.3390/md19060316 (PMC8227522; doi:10.3390/md19060316)
Supplement: Supplementary file 1 [file marinedrugs-19-00316-s001.zip › marinedrugs-1222583-supplementary.pdf]

Supplementary

Mining Indonesian microbial biodiversity for novel natural compounds by a combinatory genome mining and molecular networking approach

Ira Handayani [1,2,a], Hamada Saad [3,8,a], Shanti Ratnakomala [2], Puspita Lisdiyanti [2], Wien Kusharyoto [2], Janina Krause[1], Wolfgang Wohlleben[1], Andreas Kulik[1], Saefuddin Aziz [3], Harald Gross [3], Athina Gavriilidou [4], Nadine Ziemert [4,5], and Yvonne Mast[1,5,6,7,*]

[1] Department of Microbiology/Biotechnology, Interfaculty Institute of Microbiology and Infection Medicine, Tübingen (IMIT), Cluster of Excellence 'Controlling Microbes to Fight Infections', University of Tübingen, Auf der Morgenstelle 28, 72076 Tübingen, Germany; e-mail@e-mail.com

[2] Research Center for Biotechnology, Indonesian Institute of Sciences (LIPI), Jl. Raya Jakarta-Bogor KM.46, Cibinong, West Java 16911, Indonesia; p2biotek@bioteknologi.lipi.go.id.

[3] Department of Pharmaceutical Biology, Institute of Pharmaceutical Sciences, University of Tübingen, Auf der Morgenstelle 8, 72076 Tübingen, Germany; e-mail@e-mail.com

[4] Applied Natural Products Genome Mining, Interfaculty Institute of Microbiology and Infection Medicine Tübingen (IMIT), Cluster of Excellence 'Controlling Microbes to Fight Infections', University of Tübingen, Auf der Morgenstelle 28, 72076 Tübingen, Germany.; e-mail@e-mail.com

[5] German Center for Infection Research (DZIF), Partner Site Tübingen, Tübingen, Germany

[6] Department of Bioresources for Bioeconomy and Health Research, Leibniz Institute DSMZ – German Collection of Microorganisms and Cell Cultures, Inhoffenstraße 7B, 38124 Braunschweig, Germany; e-mail@e-mail.com

[7] Department of Microbiology, Technical University of Braunschweig, Braunschweig, Germany; e-mail@e-mail.com

[8] Department of Phytochemistry and Plant Systematics, Division of Pharmaceutical Industries, National Research Centre, Dokki, Cairo (Egypt)

[a] Ira Handayani and Hamada Saad contributed equally to this work

* Correspondence: yvonne.mast@dsmz.de; Tel.: +49-531-2616-358

Preparation of test plates for agar diffusion tests

A. Preparation of *Bacillus subtilis* test plates

B. subtilis is grown for 4 to 7 days at a temperature of 29°C in sporulation medium ($FeCl_3$ x $6H_2O$ 1 mg, $MnCl_2$ x $2xH_2O$ 14.5 mg, NH_4Cl 540 mg, Na_2SO_4 105 mg, KH_2PO_4 87 mg, $CaCl_2$ 194.7 mg, NH_4NO_3, $MgCl_2$ x $6H_2O$ 8.3 mg, glucose 2 g, Na-L-glutamate x H_2O 1.9 g, pH7.1). The *B. subtilis* culture was harvested (10 min, 5000 rpm, 4 ° C) and twice washed with 20 mL of $H_2O_{deion.}$. The washed pellets were dissolved with 15 mL H_2O_{deion}. and heated for 30 minutes at 70 °C to kill remaining vegetative cells. The spore suspension can be stored for a long time at a temperature not exceeding 4 ° C. For *B. subtilis* test plates, 10 mL/L of the spore suspension were poured into *B. subtilis* test agar (KH_2PO_4 3g, K_2HPO_4 7g, tri-Na citrate.2H2O 0.5 g, MgSO4 x 7H2O 0.1 g, (NH4)2SO4 1g). Plates were incubated overnight at 37 °C

B. Preparation of *M. luteus* and *P. fluorescens* test plates

Bioassays test plates for *M. luteus* and *P. fluorescens* were prepared by spread *M. luteus* or *P. fluorescens* cells evenly over YM agar plates surface. The plates were used directly for inhibition zone tests. Plates were incubated overnight at 28°C.

C. Preparation of *E. coli* and *S. carnosus* test plates

In liquid LB agar, cells of an overnight culture of *E. coli* of *S. carnosus* were poured in a ratio of 1: 100 and pipetted into petri dishes. For bioassays, the plates were incubated overnight at 37°C.

Polyphasic characteristics of the most closely related type strains of each clade A-D shown in Figure 3

Clade A – *Streptomyces luteus* (according to Luo et al., 2017 [1]):

The strain was aerobic, Gram-stain-positive, with an optimum NaCl concentration for growth of 5% (w/v). The isolate formed white aerial mycelium that was long filamentous with few branches; the substrate mycelium possessed long, smooth-surfaced spore chains bearing smooth spores and produced a yellow diffusible pigment. The strain contained iso-C16:0, anteiso-C15:0, anteiso-C17:0 and C16:0 as major

cellular fatty acids. The predominant menaquinones of the strain were MK-9(H6), MK-9(H4) and MK-9(H10). The whole-cell sugar pattern contained glucose and ribose. The polar lipid pattern of the strain consisted of phosphatidylethanolamine, diphosphatidylglycerol, phosphatidylinositol, phosphatidylglycerol and phosphatidylinositolmannosides.

Clade B – *Streptomyces capillispiralis* (according to Mertz and Higgins et al., 1982 [2]):

Streptomyces capillispiralis produces a cephalosporin C-4 carboxymethyl esterase. The key characteristics of this species are gray spore mass color, spiral spore chains, and hairy spores.

Clade C – *Streptomyces bungoensis* (according to Eguchi et al., 1993 [3]):

Streptomyces bungoensis forms gray aerial mass, spiral spore chains, and a spiny spore surface; forms a melanoid pigment on tyrosine agar, on peptone-yeast extract-iron agar, and in tryptone-yeast extract broth; and has cell wall chemotype I.

Clade D – *Streptomyces spongiicola* (according to Huang et al., 2016 [4]):

The major menaquinones were MK-9 (H6) (65.6 %), MK-9 (H4) (23.8 %) and MK-9 (H8) (10.6 %). The predominant fatty acids were anteiso-C15 : 0 (25.5 %), iso-C16 : 0 (19.5 %) and iso-C15 : 0 (15.4 %). The predominant phospholipids were diphosphatidylglycerol, phosphatidylglycerol and phosphatidylethanolamine. In addition, four unidentified phospholipids were found. The G+C content of the genomic DNA was 69.8 mol%.

Figures and Tables

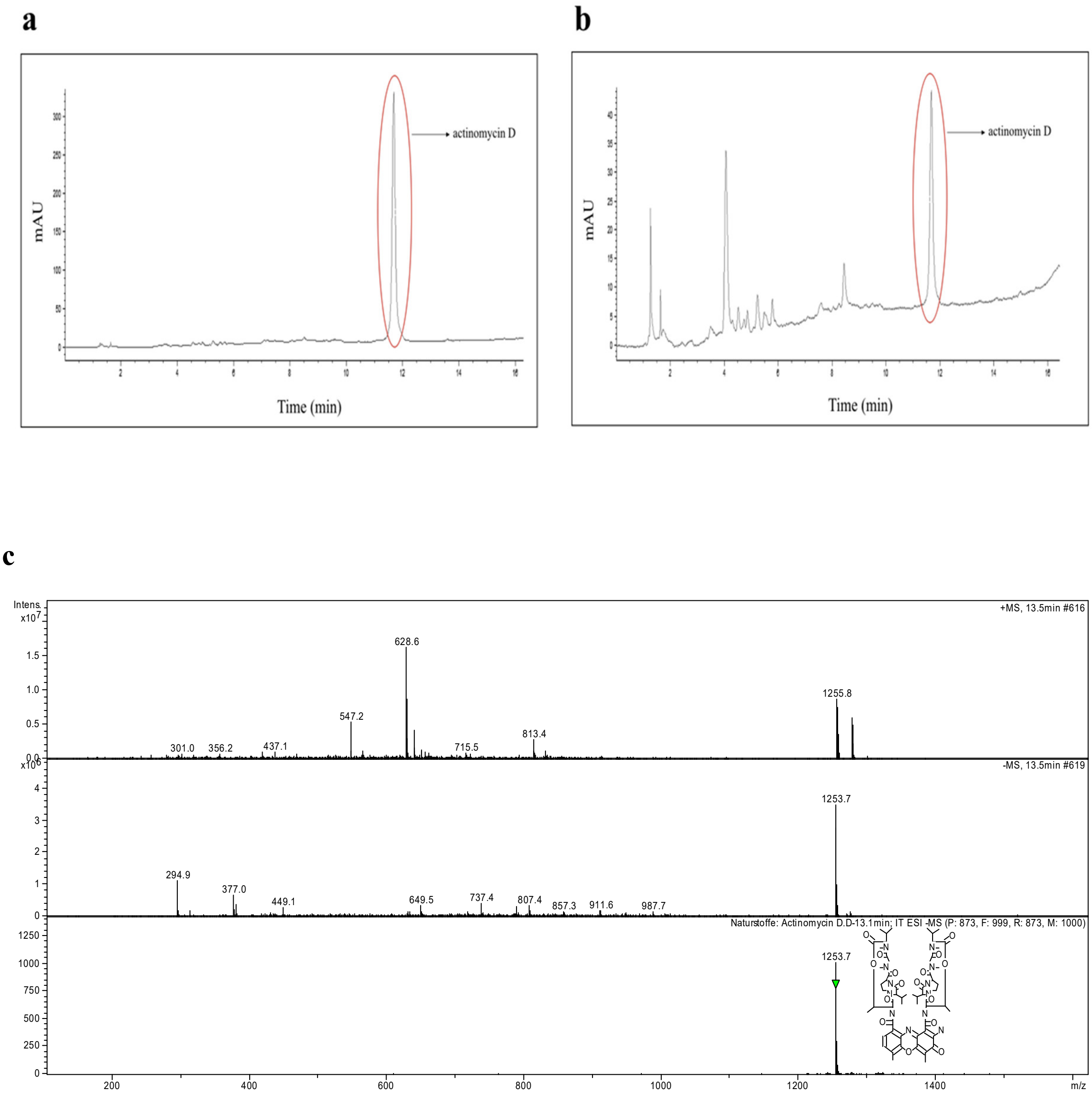

Figure S1. Actinomycin D production in DHE 6-7 (a) and DHE 5-1 (b). Peaks in HPLC representing actinomycin are marked with red colour. Mass spectra of actinomycin D detected in DHE 6-7 (c).

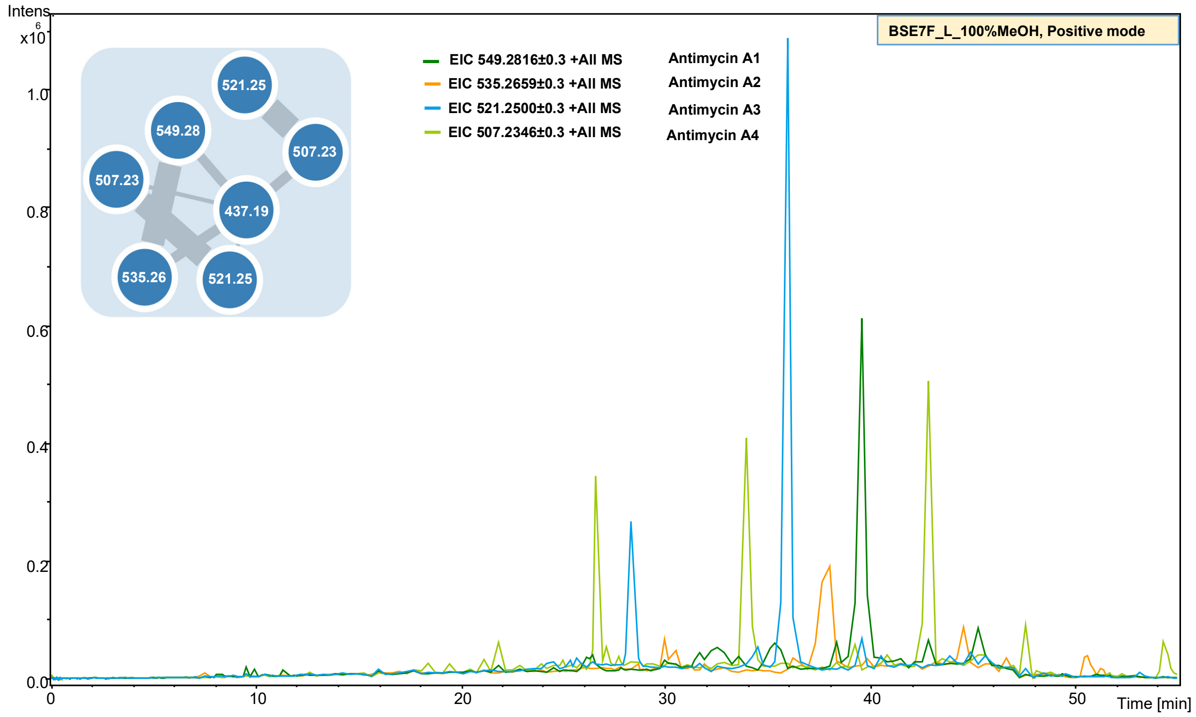

Figure S2. Positive extracted ion chromatograms (EICs), ions cluster and predicted molecular formula of antimycins.

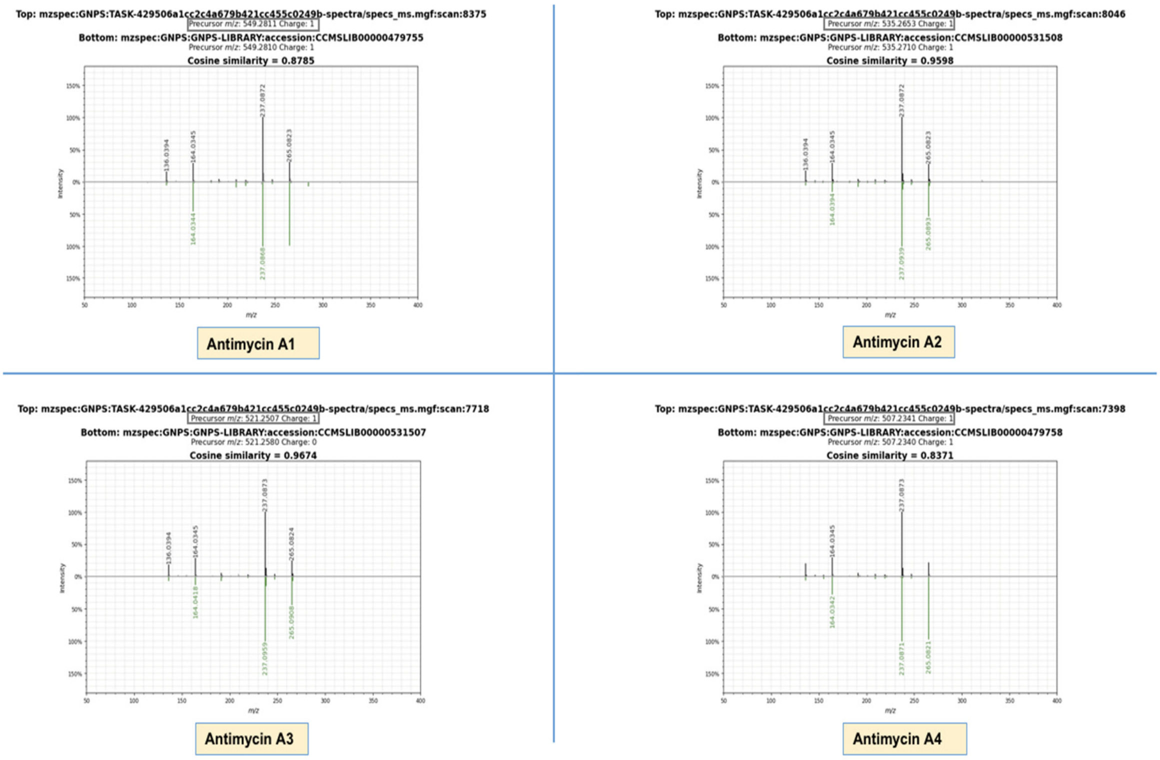

Figure S3. GNPS spectral libraries hits of antimycins.

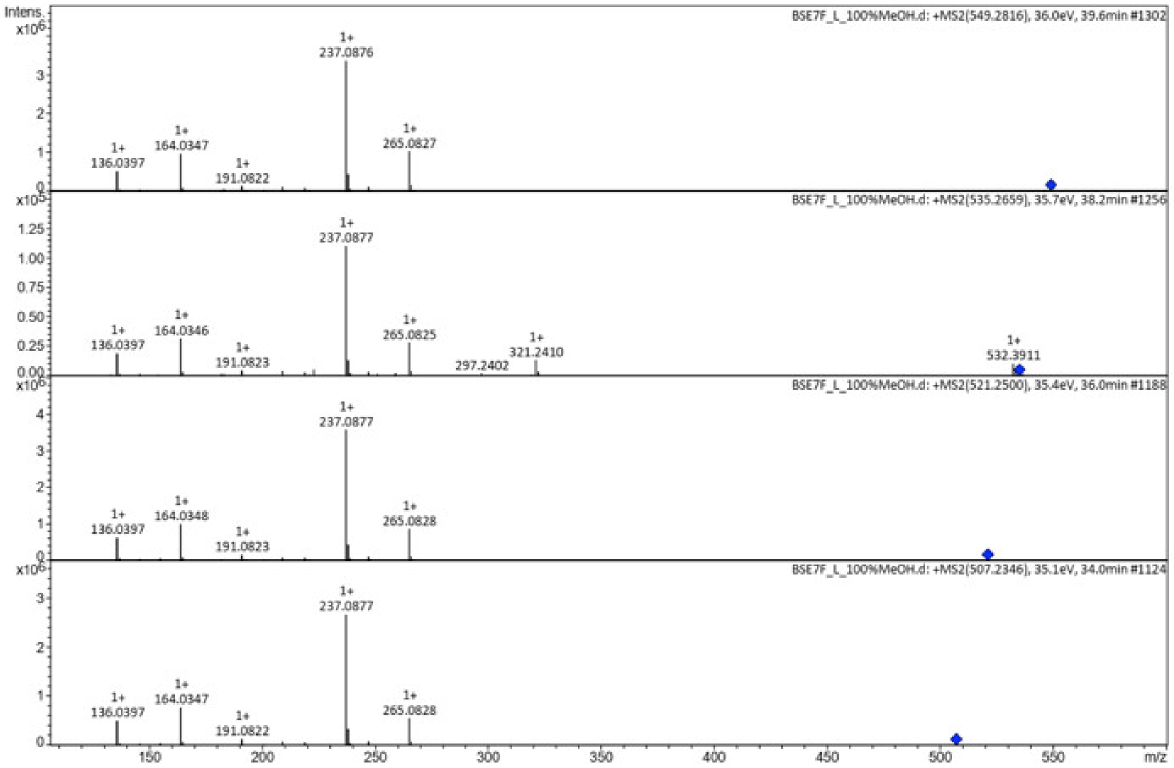

Figure S4. Positive MS^2 spectra of the detected antimycins from isolate BSE 7F.

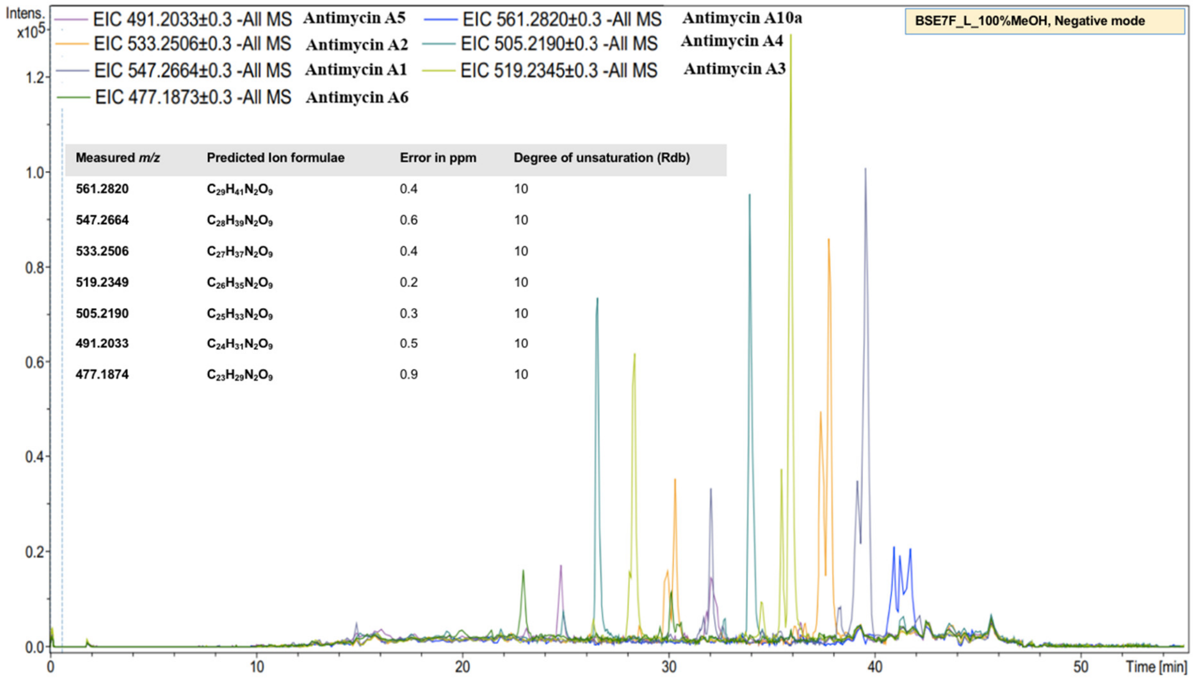

Measured *m/z*	Predicted Ion formulae	Error in ppm	Degree of unsaturation (Rdb)
561.2820	$C_{29}H_{41}N_2O_9$	0.4	10
547.2664	$C_{28}H_{39}N_2O_9$	0.6	10
533.2506	$C_{27}H_{37}N_2O_9$	0.4	10
519.2349	$C_{26}H_{35}N_2O_9$	0.2	10
505.2190	$C_{25}H_{33}N_2O_9$	0.3	10
491.2033	$C_{24}H_{31}N_2O_9$	0.5	10
477.1874	$C_{23}H_{29}N_2O_9$	0.9	10

Figure S5. Negative EICs and predicted molecular formulae of antimycins.

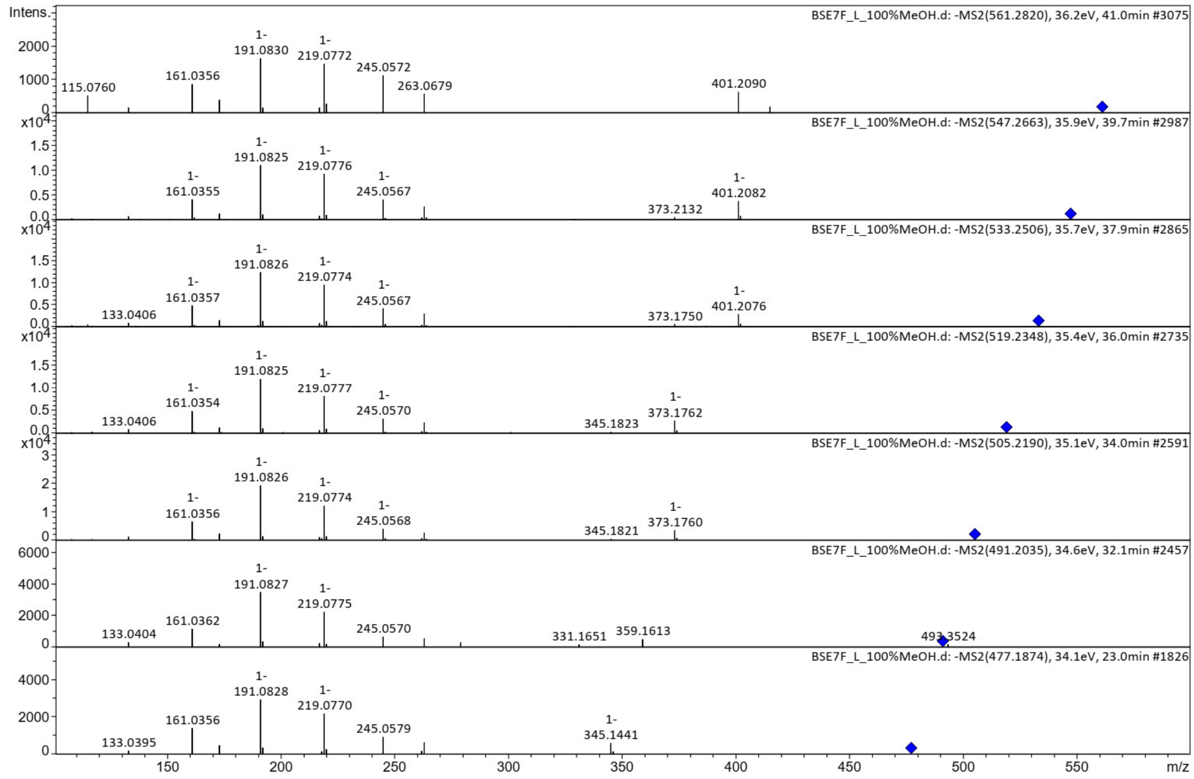

Figure S6. Negative MS^2 spectra of the detected antimycins from isolate BSE 7F.

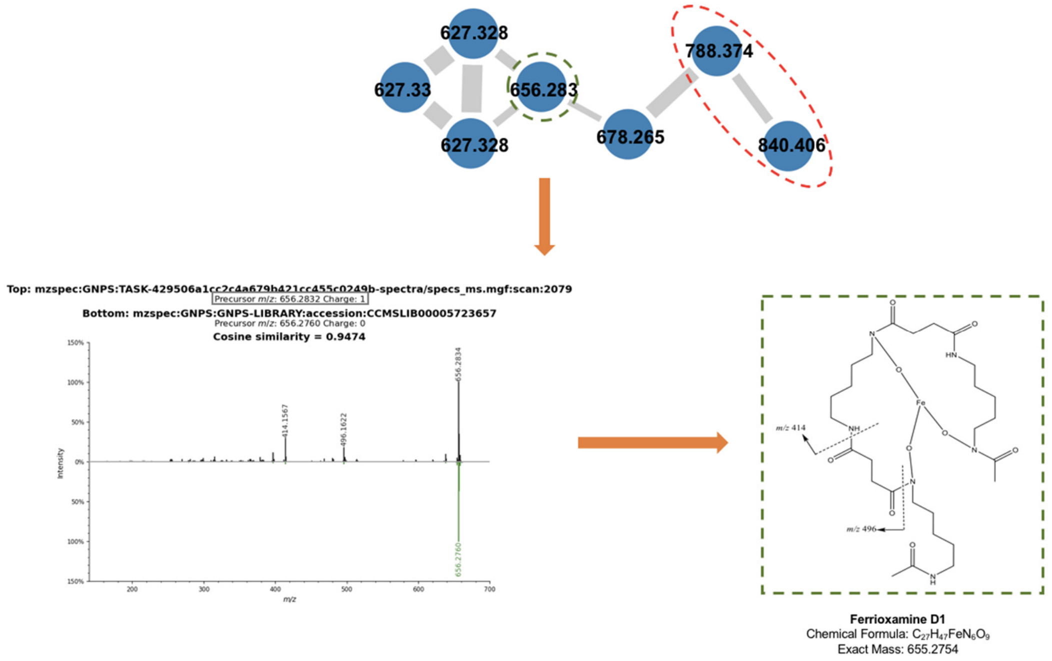

Figure S7. Ions cluster of ferrioxamines and GNPS spectral libraries hit of ferrioxamine D1.

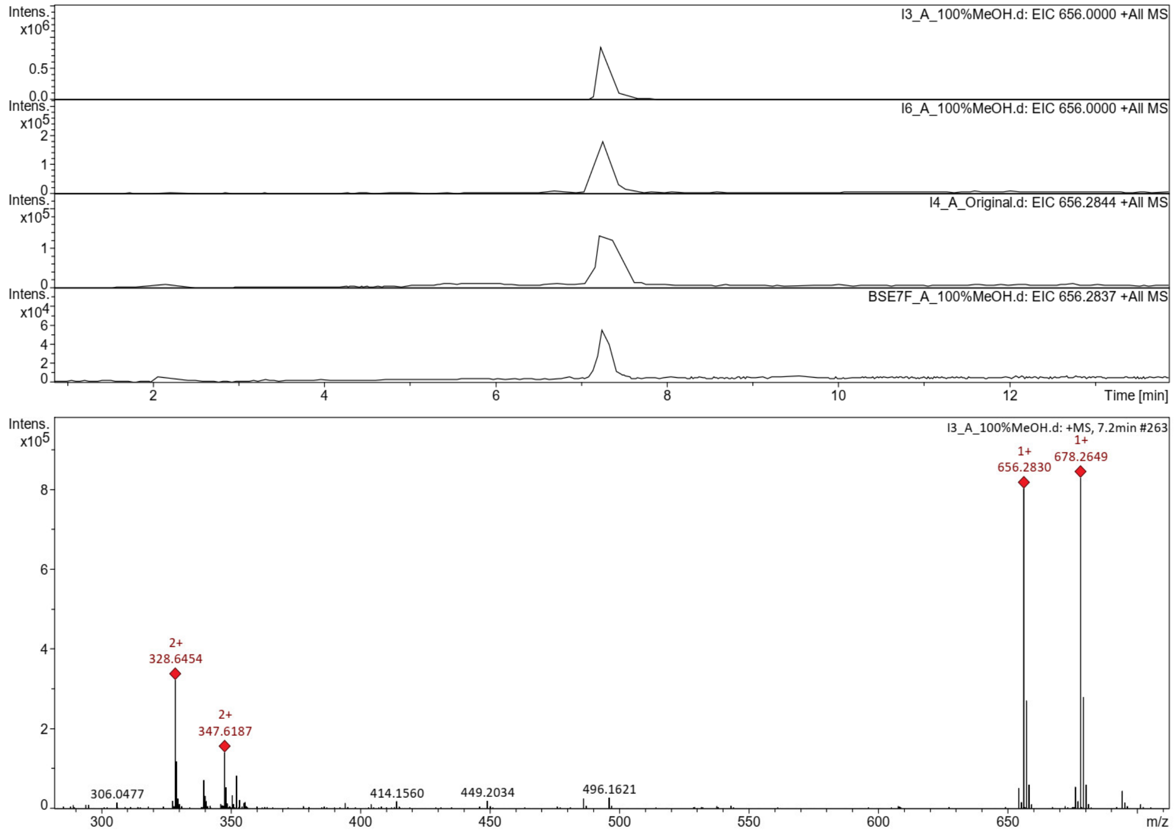

Figure S8. Positive EICs and molecular formula prediction of ferrioxamine D1.

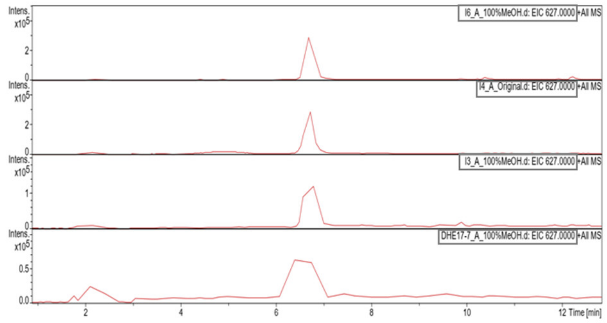

Measured *m/z*	Predicted Ion formulae	Error in ppm	Degree of unsaturation (Rdb)
**627.3303**	**$C_{26}H_{51}FeN_8O_6$**	4.3	6

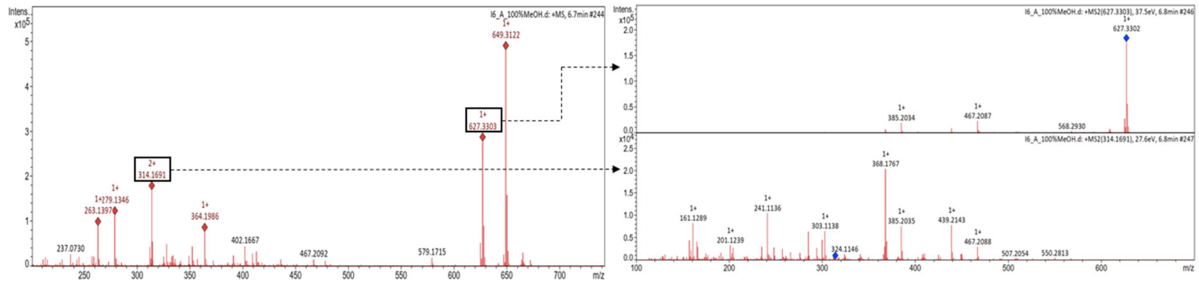

Figure S9. Positive EICs, molecular formula prediction and MS^2 of an unknown ferrioxamine.

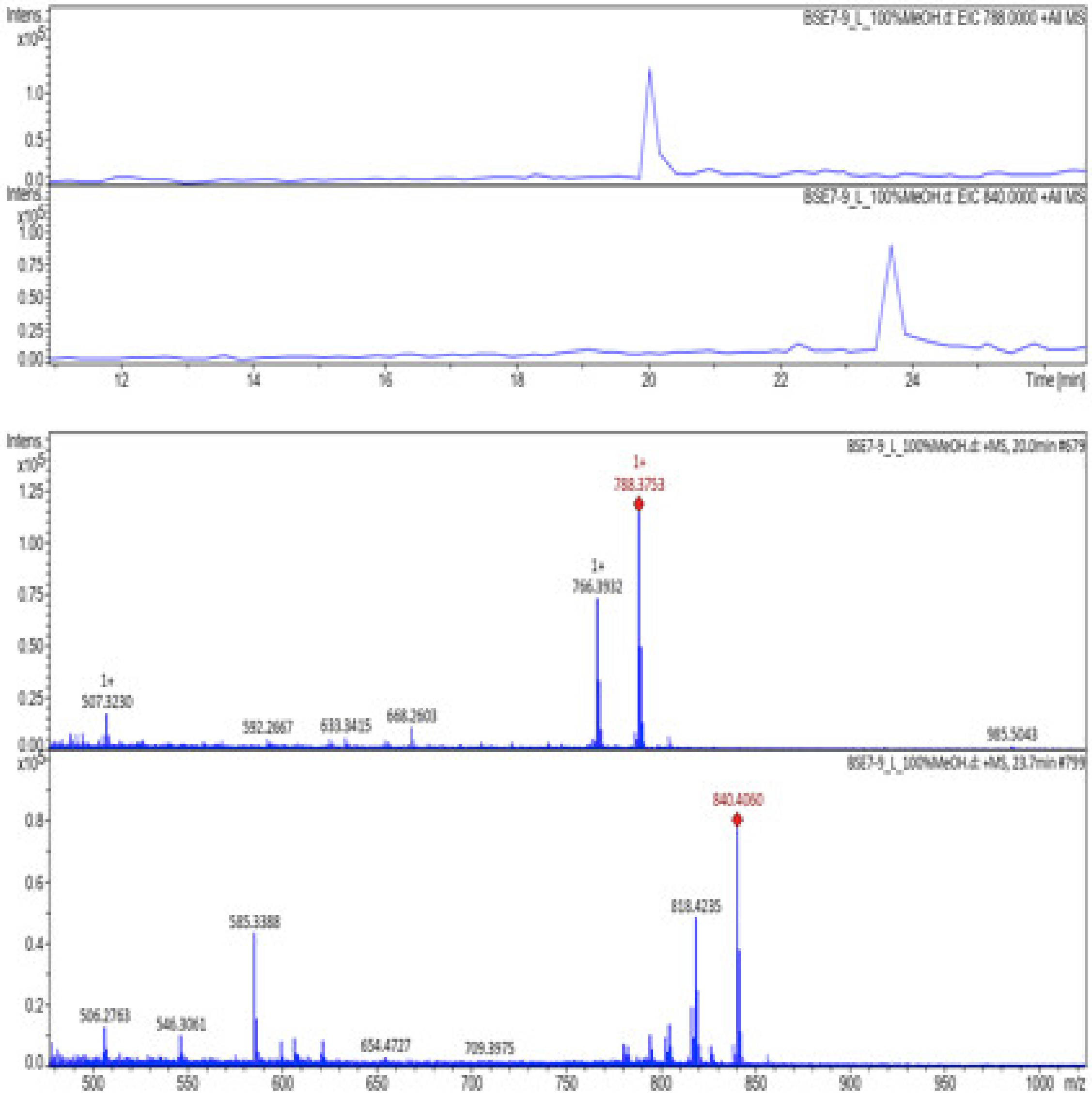

Figure S10. Positive EICs and MS[1] of unknown amphiphilic ferrioxamines.

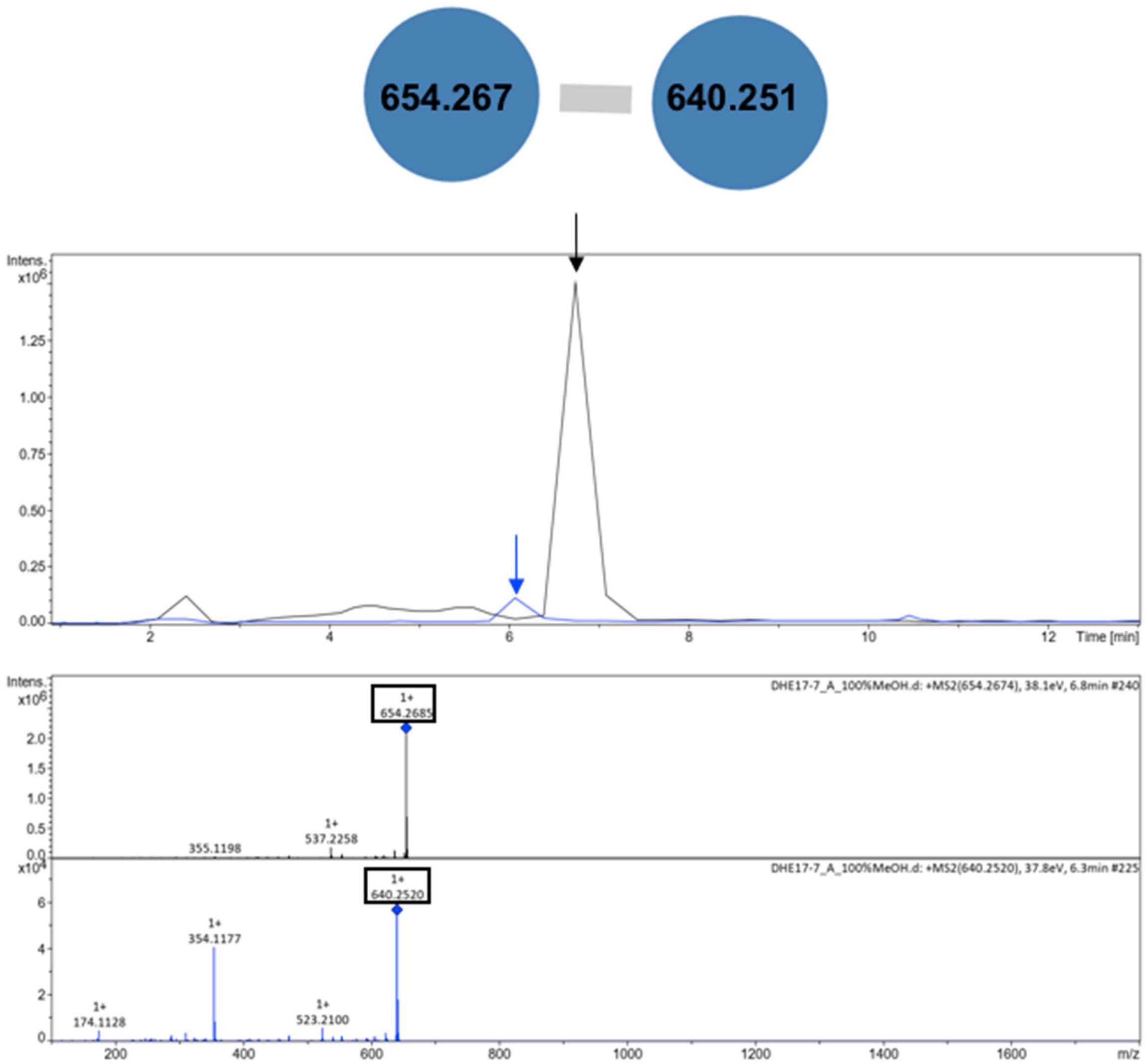

Figure S11. Positive EICs and MS^2 of DHE 17-7 ferrioxamines.

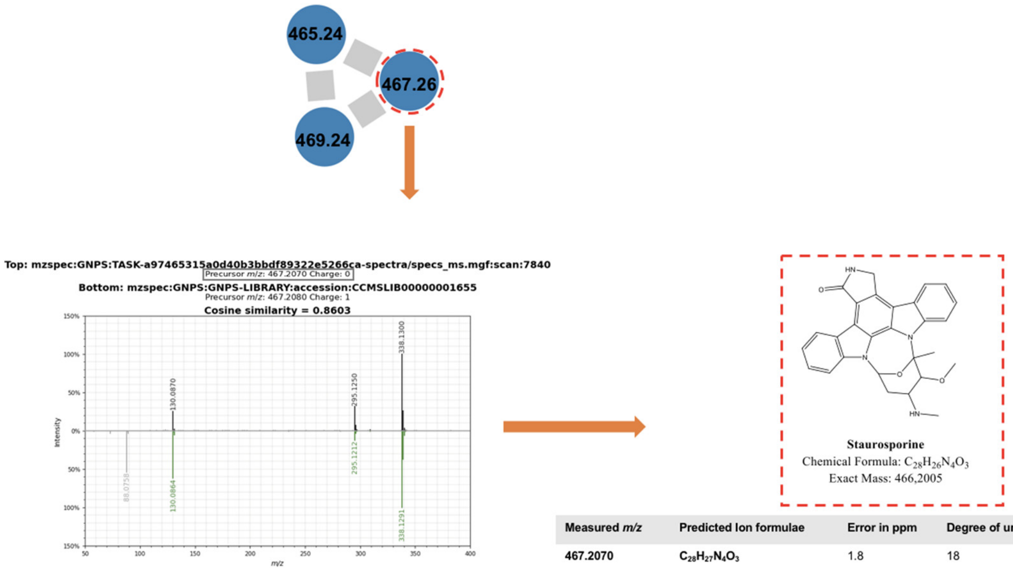

Measured *m/z*	Predicted Ion formulae	Error in ppm	Degree of unsaturation (Rdb)
467.2070	$C_{28}H_{27}N_4O_3$	1.8	18

Figure S12. Ions cluster of staurosporines and GNPS spectral libraries hit of staurosporine.

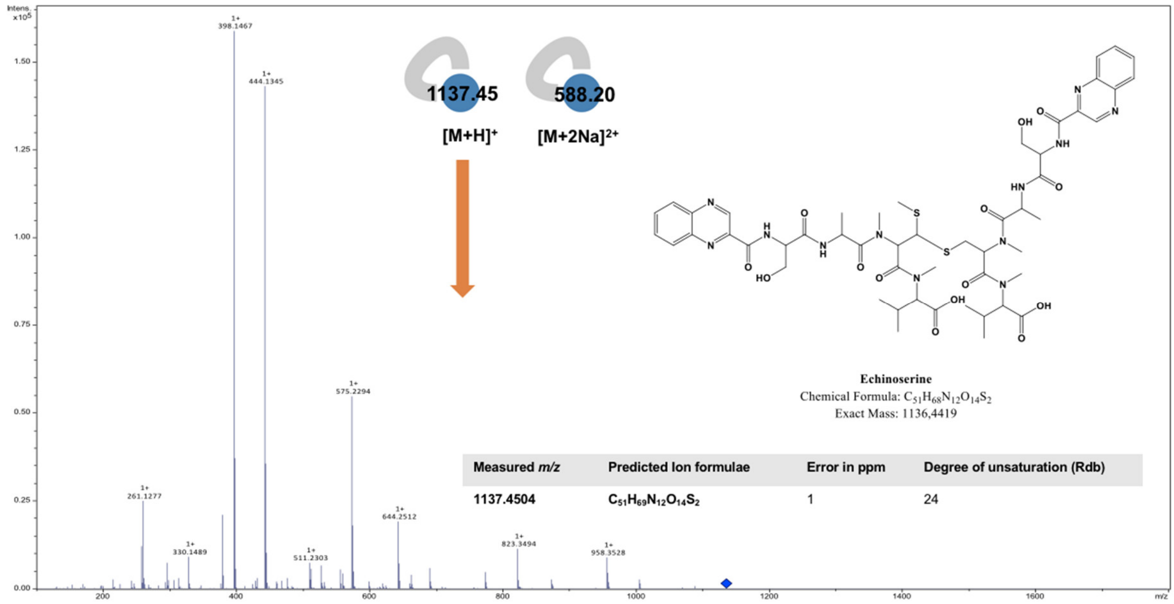

Measured *m/z*	Predicted Ion formulae	Error in ppm	Degree of unsaturation (Rdb)
1137.4504	$C_{51}H_{69}N_{12}O_{14}S_2$	1	24

Figure S13. Ions clusters of echinoserine.

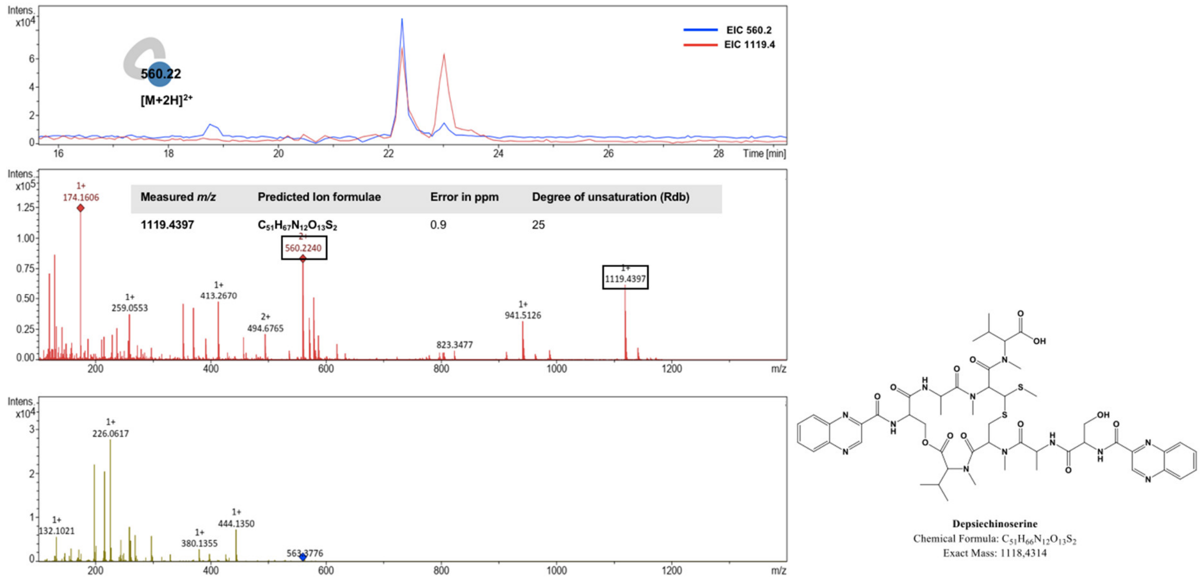

Figure S14. Ion singleton of depsiechinoserine, positive EICs and MS^2 of depsiechinoserine.

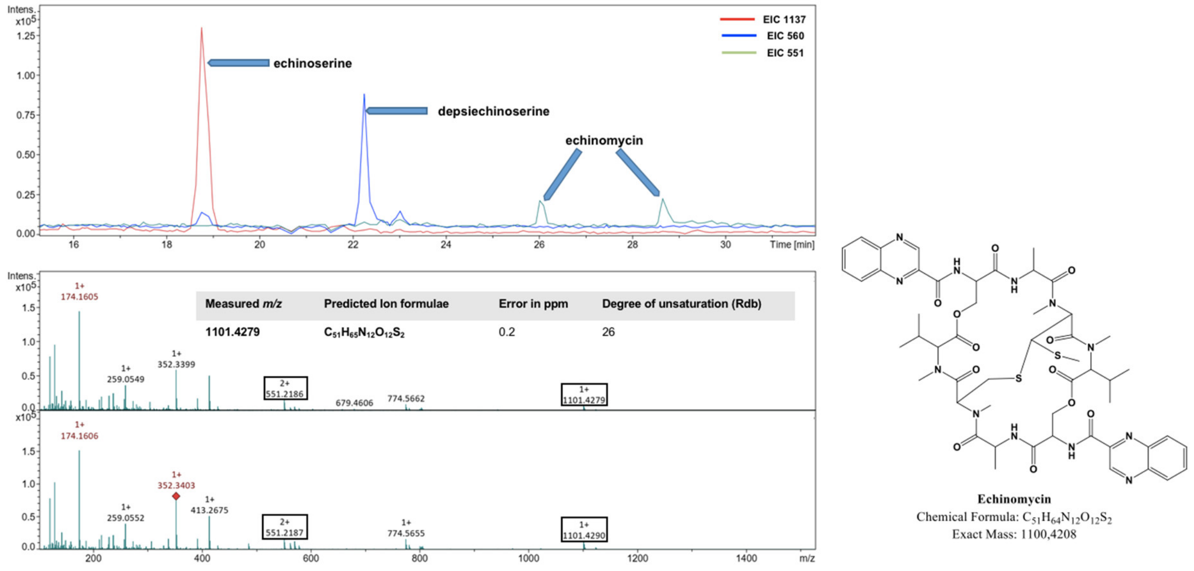

Figure S15. Positive EICs and MS^1 of echinomycin.

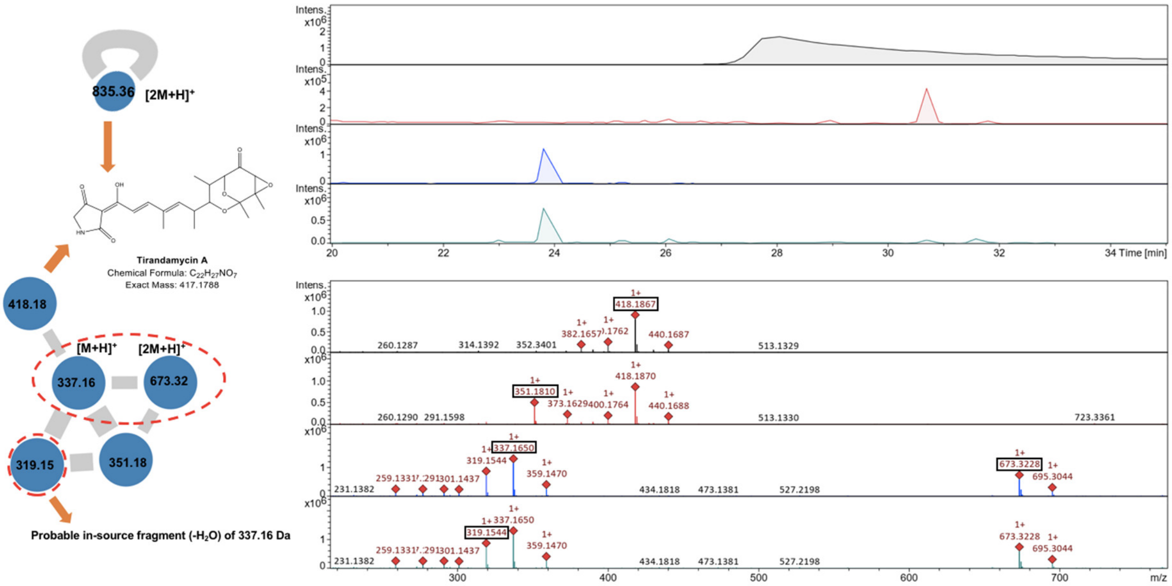

Figure S16. Ions cluster, Positive EICs and MS[1] of tirandamycin A in addition to its congeners.

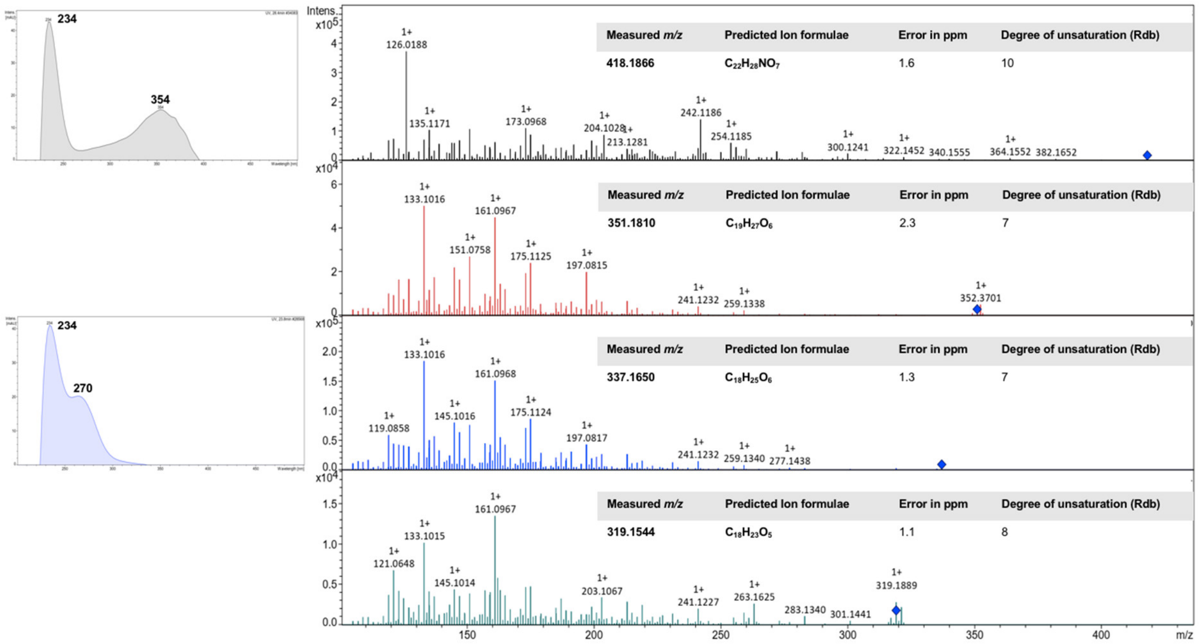

Figure S17. UV absorbance, and MS[2] of tirandamycin A in addition to its congeners.

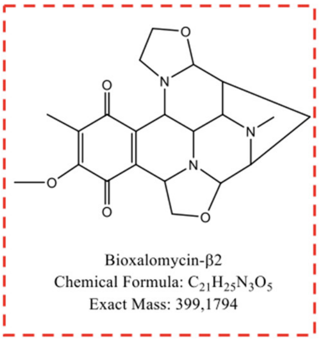

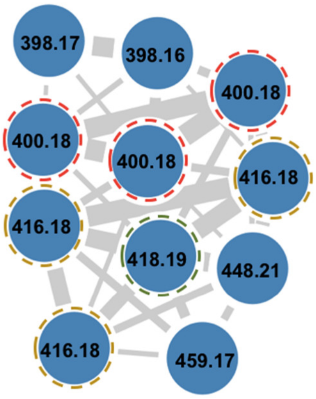

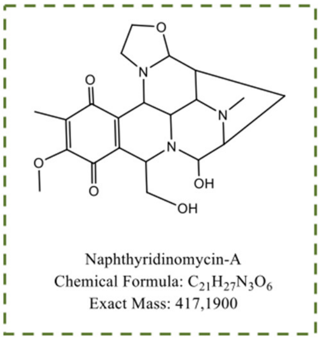

Measured *m/z*	Predicted Ion formulae	Error in ppm	Degree of unsaturation (Rdb)
459.1705	$C_{18}H_{27}N_4O_{10}$	3.6	8
448.2086	$C_{22}H_{30}N_3O_7$	1.7	10
418.1980	$C_{21}H_{28}N_3O_6$	1.9	10
416.1823	$C_{21}H_{26}N_3O_6$	1.5	11
400.1873	$C_{21}H_{26}N_3O_5$	1.0	11
398.1714	$C_{21}H_{24}N_3O_5$	0.8	12

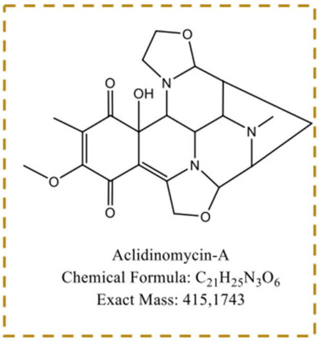

Figure S18. Ion cluster of naphthyridinomycins and their predicted molecular formula (MF).

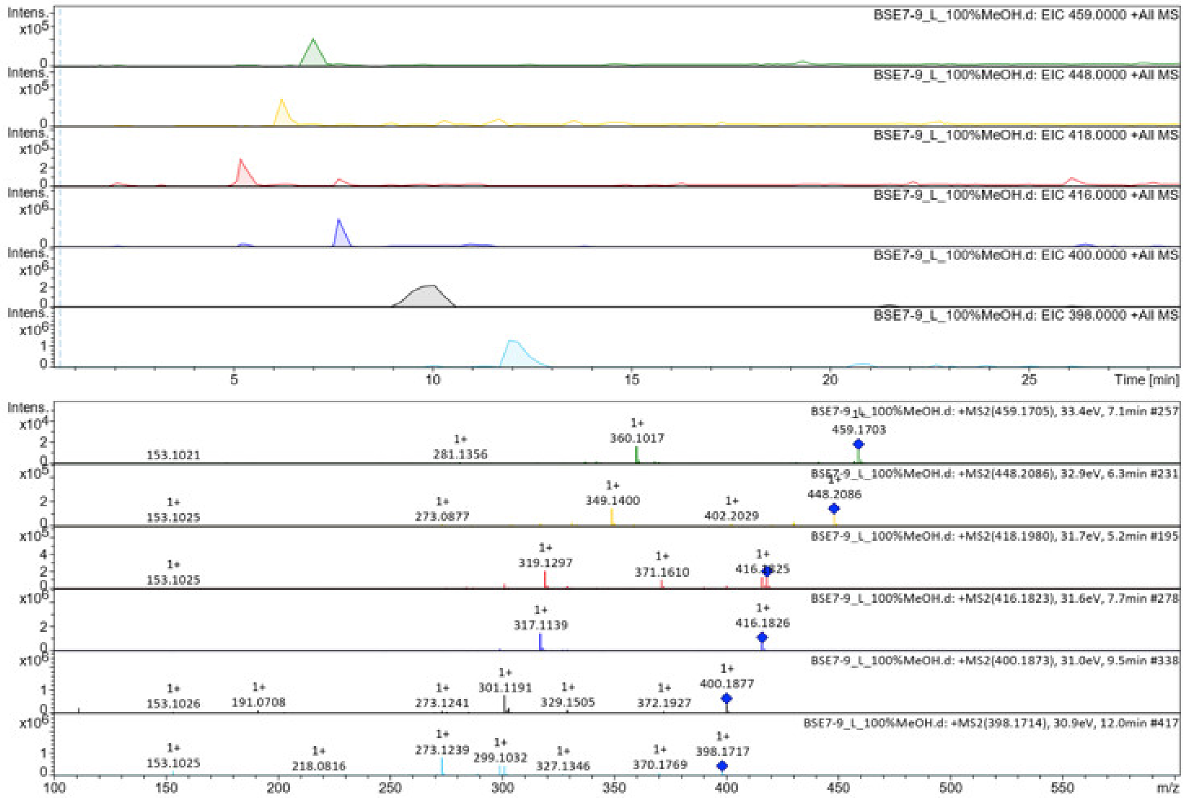

Figure S19. Positive EICs and MS^2 of naphthyridinomycin and their related entities from isolate BSE 7-9.

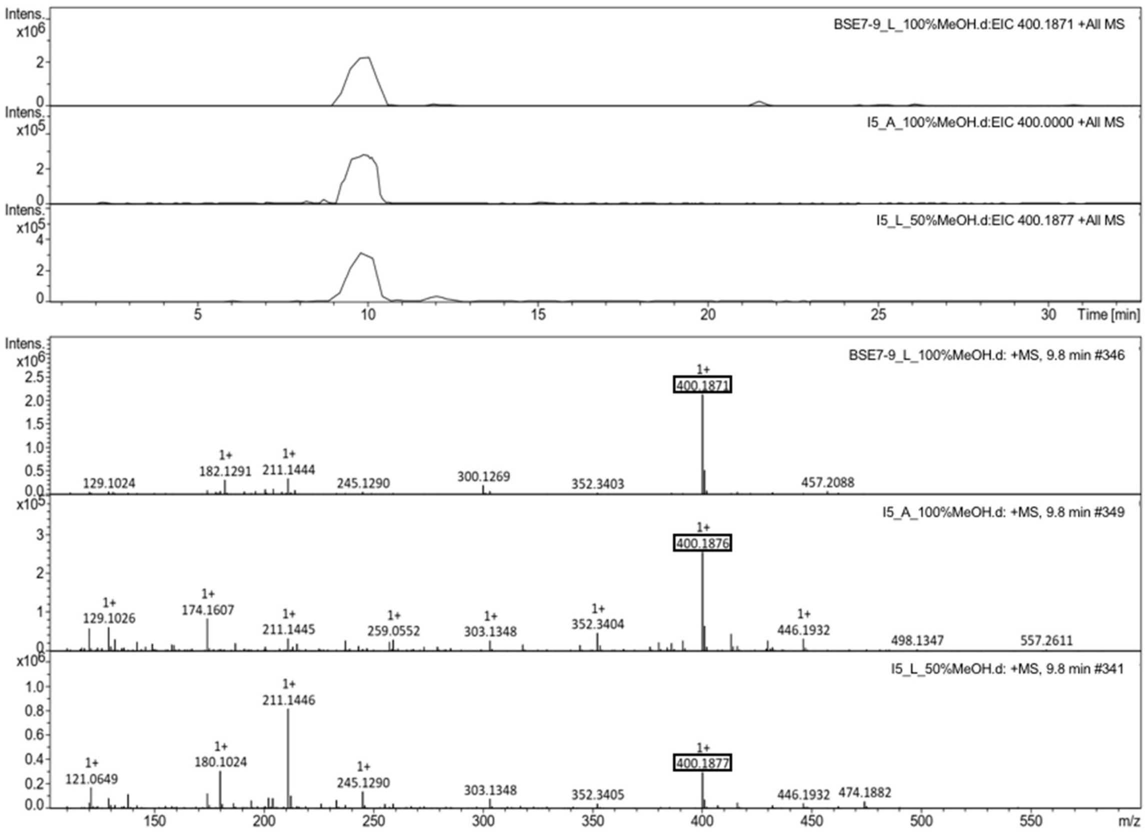

Figure S20. Positive EICs and MS[1] of naphthyridinomycin from isolates BSE 7-9 and I5.

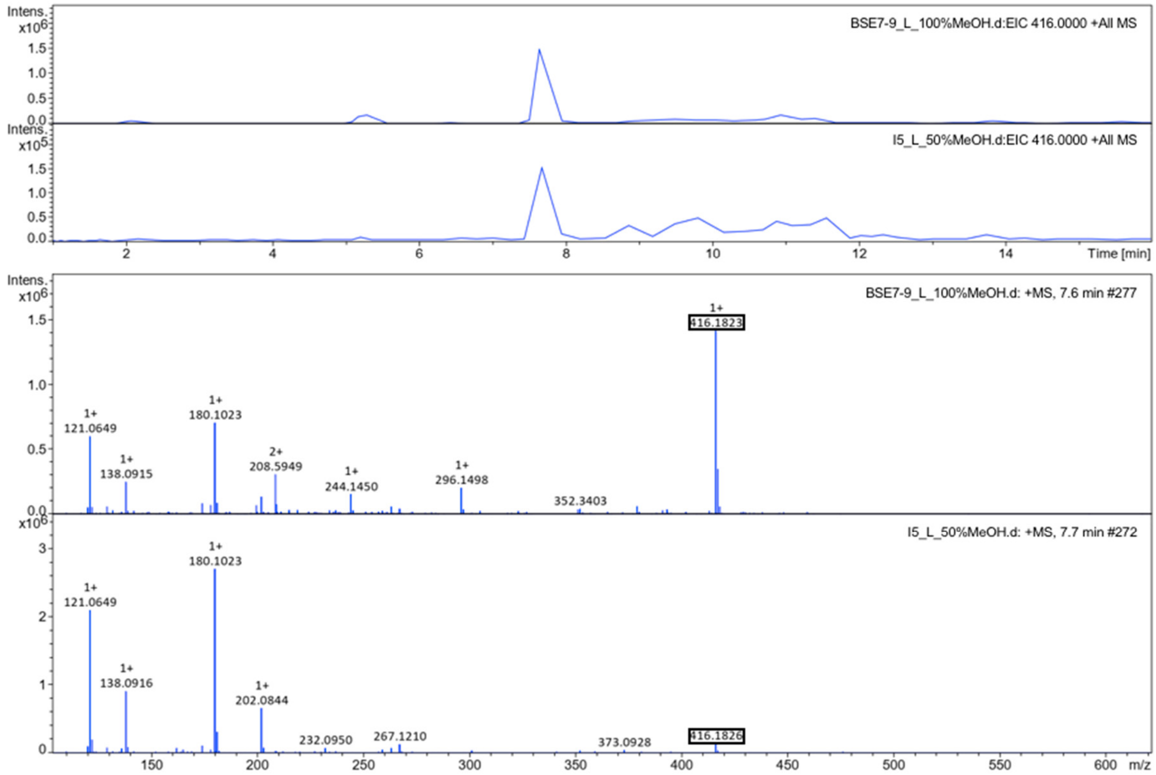

Figure S21. Positive EICs and MS[1] of aclidinomycin A from isolates BSE 7-9 and I5.

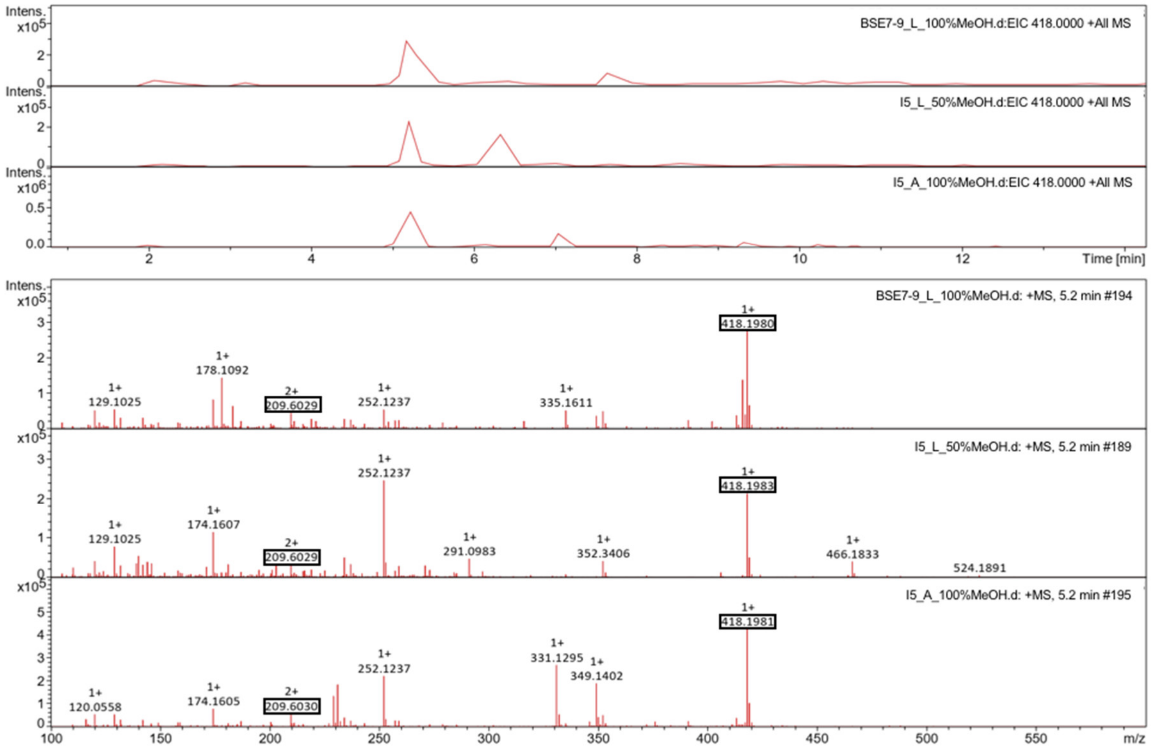

Figure S22. Positive EICs and MS[1] of bioxalomycin-β2 from isolates BSE 7-9 and I5.

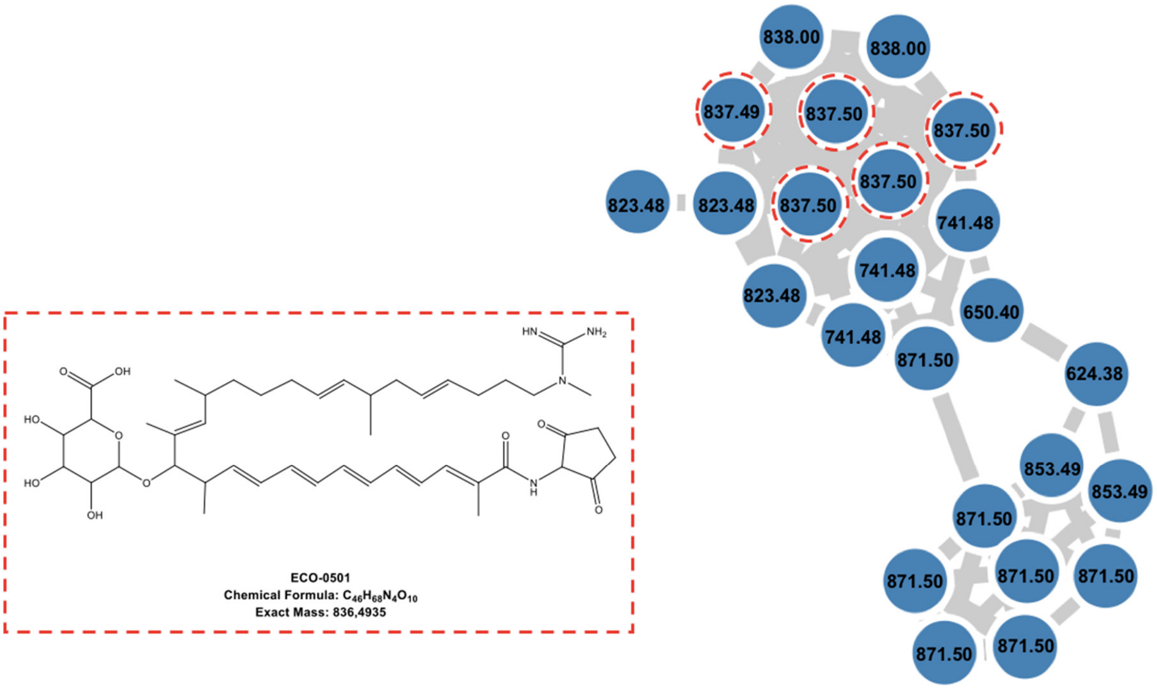

Figure S23. Ions cluster of ECO-0501 and its related congeners from isolate DHE 17-7.

Measured *m/z*	Predicted Ion formulae	Error in ppm	Degree of unsaturation (Rdb)
**871.5069**	$C_{46}H_{71}N_4O_{12}$	0.6	14
**853.4966**	$C_{46}H_{69}N_4O_{11}$	0.9	15
**837.5019**	$C_{46}H_{69}N_4O_{10}$	1.1	15
**823.4863**	$C_{45}H_{67}N_4O_{10}$	1.2	15
**741.4816**	$C_{41}H_{65}N_4O_8$	1.1	12
**650.4019**	$C_{34}H_{56}N_3O_9$	1.3	9
**624.3872**	$C_{32}H_{54}N_3O_9$	0.9	8

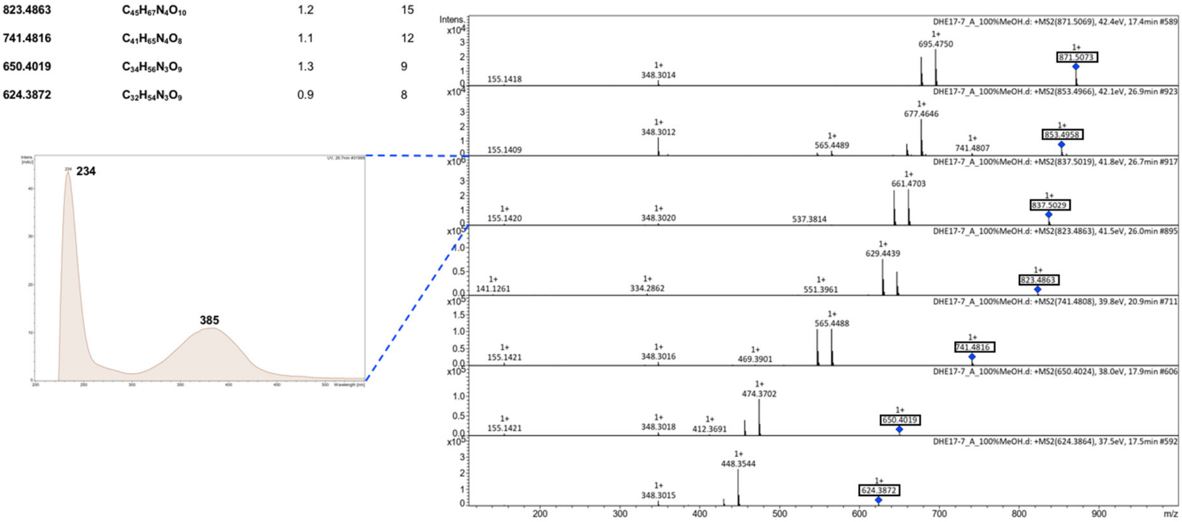

Figure S24. UV absorbance, MS2 of ECO-0501 and its related congeners from isolate DHE 17-7.

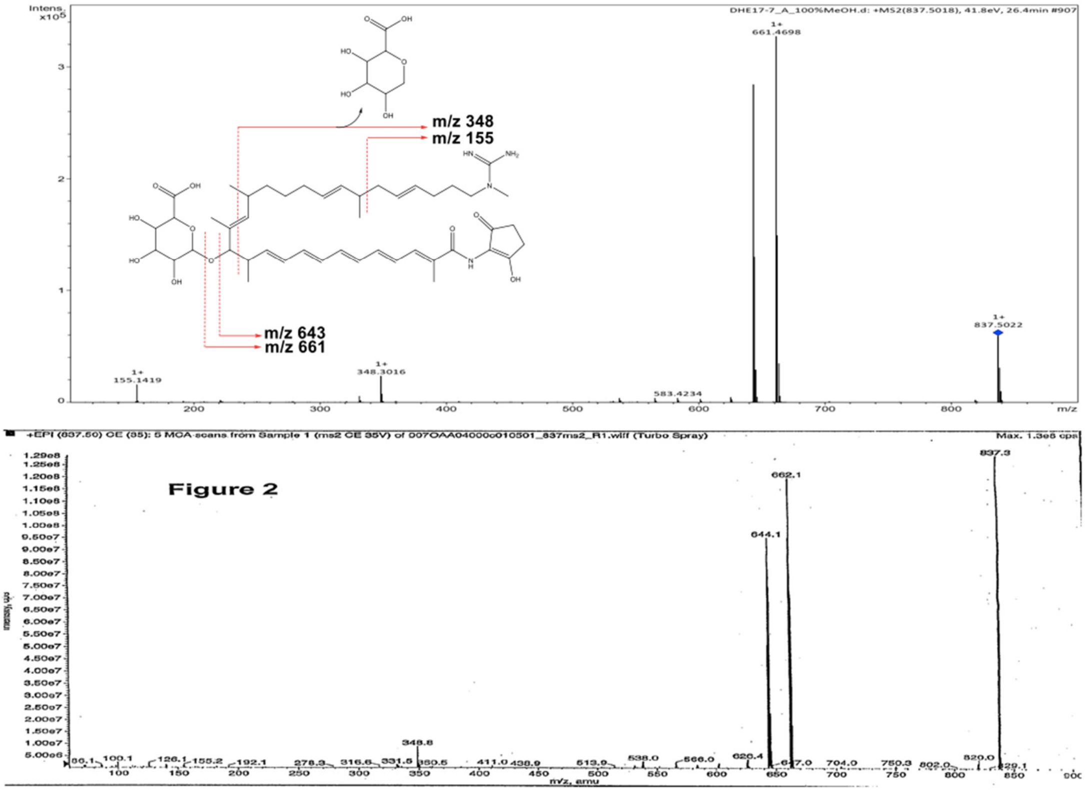

Figure S25. Comparative positive MS^2 of ECO-0501 from isolate DHE 17-7 and its reported version from *Amycolatopsis orientalis*.

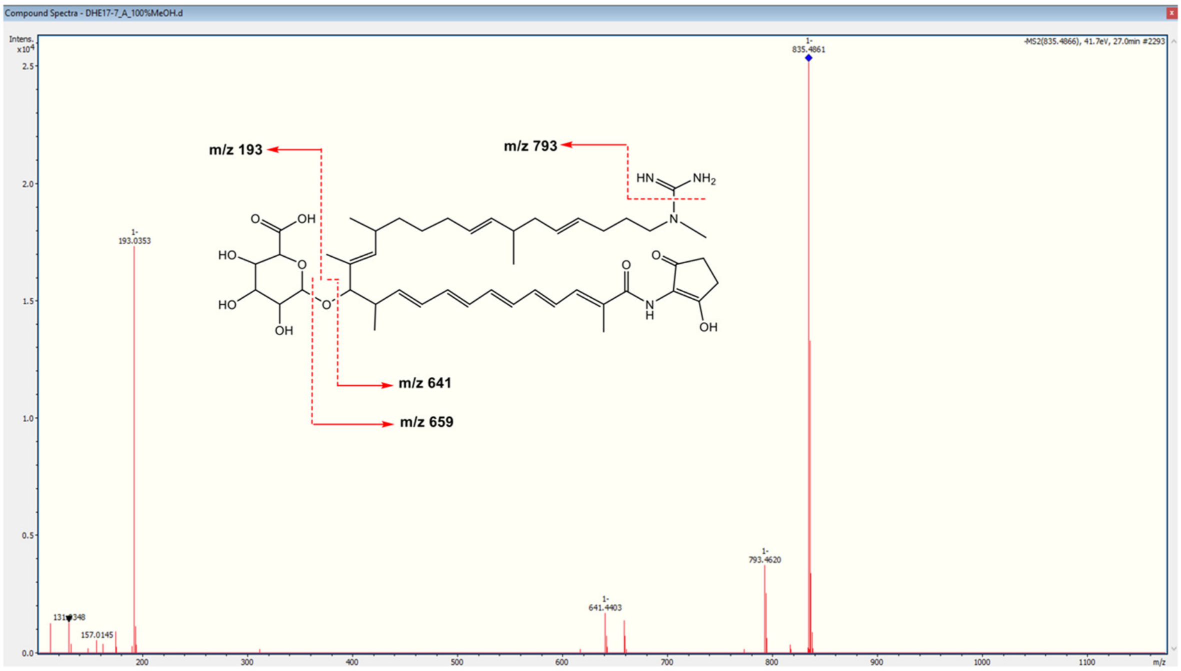

Figure S26. Negative MS2 of ECO-0501 from isolate DHE 17-7 and its proposed fragmentation scheme.

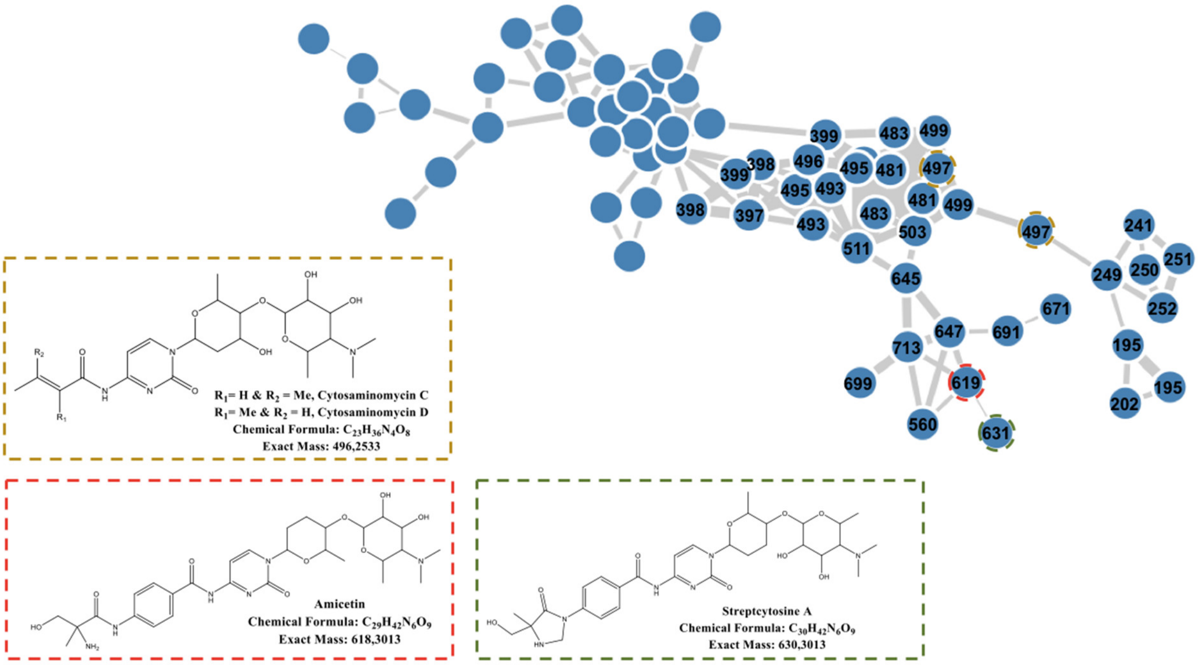

Figure S27. Ions cluster of amicetins and its related congeners from isolate SHP 22-7.

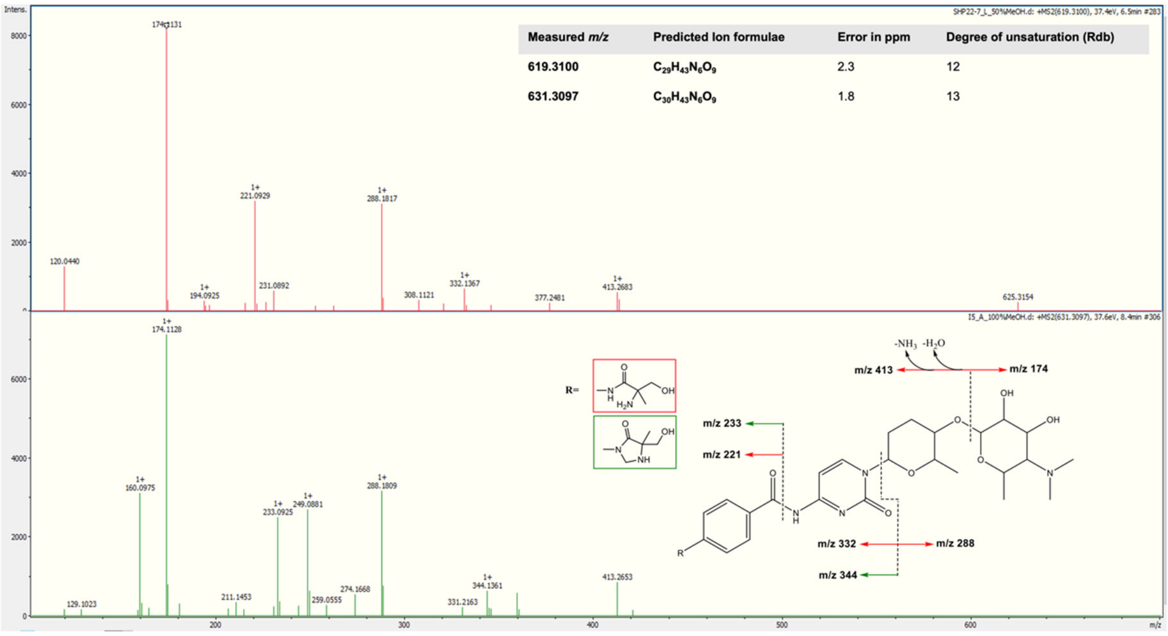

Figure 28. Comparative positive MS^2 of amicetin and streptocytosin A.

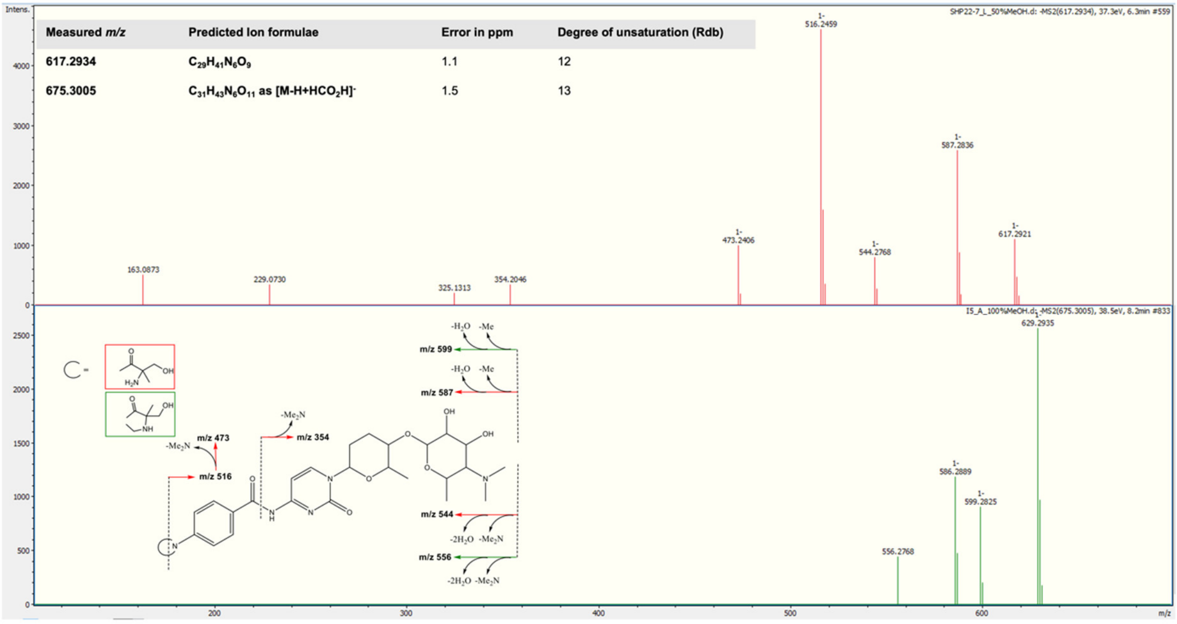

Figure S29. Comparative negative MS^2 of amicetin and streptocytosin A

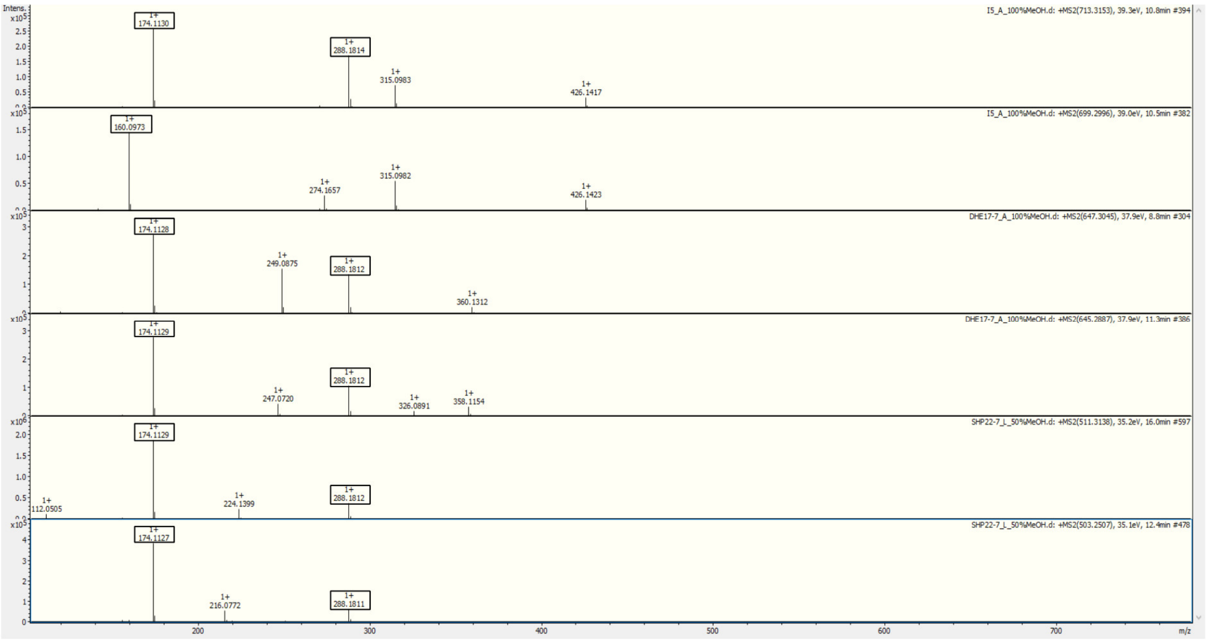

Figure S30. Comparative positive MS2 of some unknown members of amicetin molecular family.

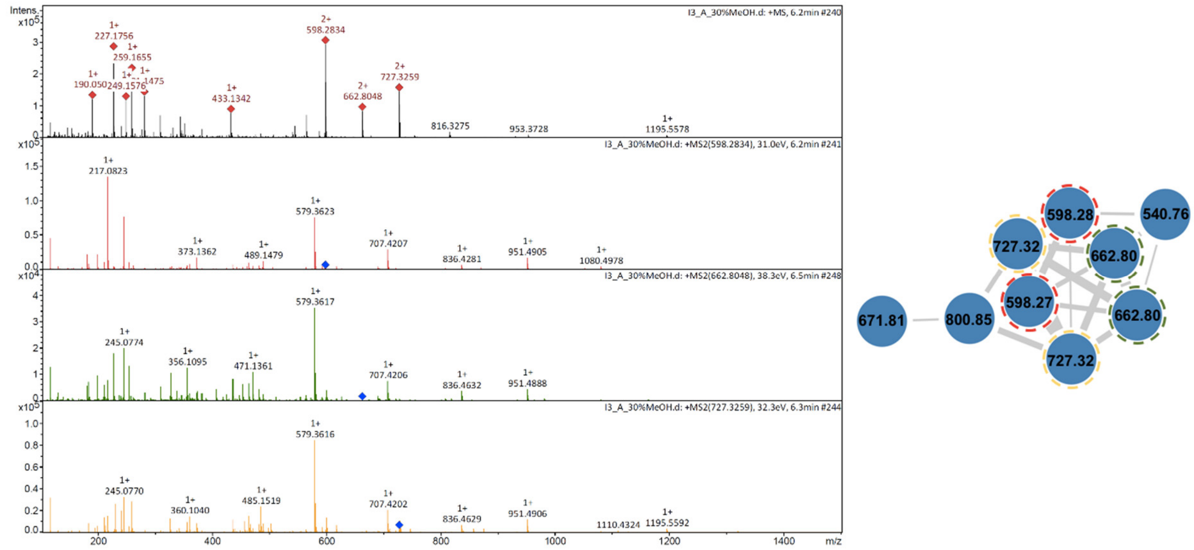

Figure S31. Comparative positive MS^2 of some unknowns of likely peptides.

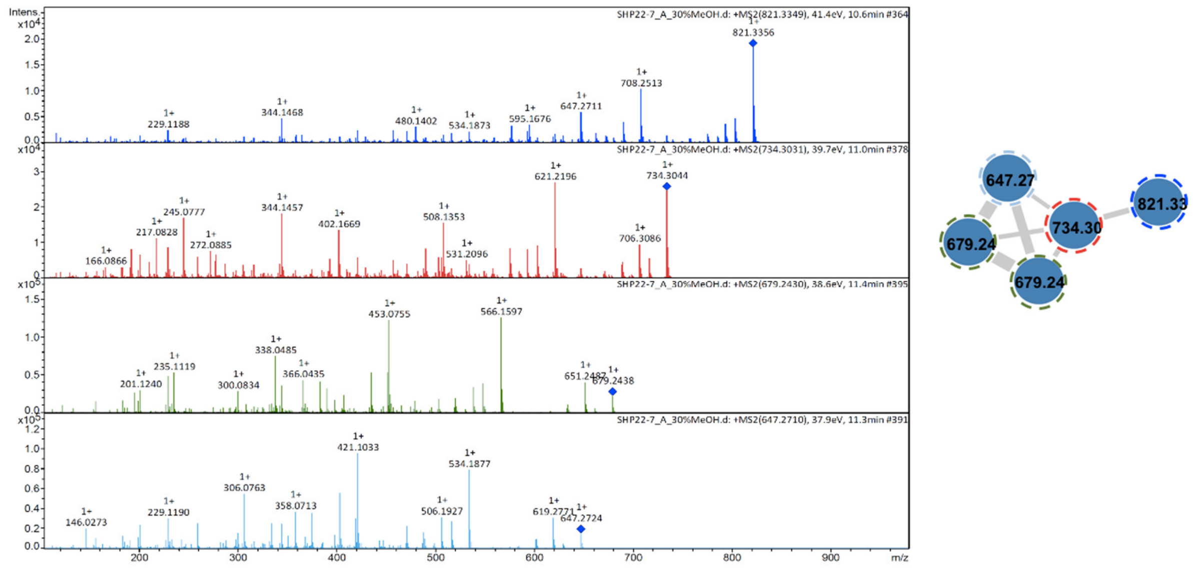

Figure S32. Comparative positive MS^2 of compound group I from isolate SHP 22-7.

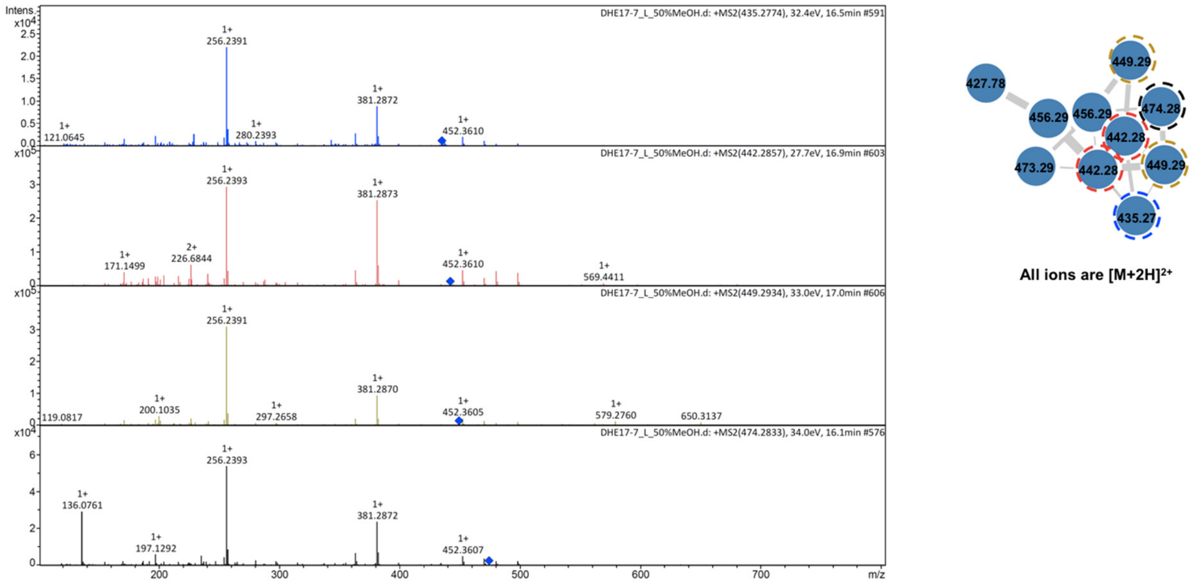

Figure S33. Comparative positive MS^2 of compound group II from isolate DHE 17-7

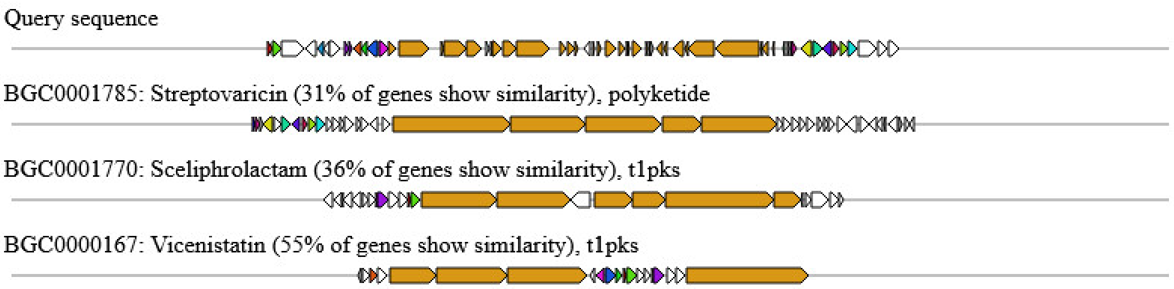

Figure S34. Cluster similarity between the DHE 17-7 gene region 24 (query sequence) and the streptovaricin, sceliphrolactam and vicenistatin cluster.

Table S1. Genome characteristics from nine Indonesian actinomycetes strain isolates.

Strain	Genome size (bp)	Contigs	GC content (%)	Coding sequence (CDS)	Transfer RNA (tRNA)	Ribosomal RNA (rRNA)
DHE 17-7	8,364,241	5	72.11	7,895	83	15
DHE 7-1	8,144,351	3	72.08	8,168	90	18
SHP 22-7	7,899,734	146	72.20	4,602	63	18
14	7,712,674	7	72.26	6,877	84	18
I3	7,638,058	7	72.26	7,394	83	18
I5	7,611,235	4	72.27	6,842	80	18
BSE 7F	7,510,161	5	72.30	6,880	79	21
BSE 7-9	7,435,252	5	72.29	6,562	82	18
I6	7,054,598	2	72.47	6,005	79	18

Table S2. media tested for antibiotic production in agar and liquid culture. All data refer to 1 l $H_2O_{deion.}$. For solid media 16 g/l agar is added, except for R5 medium 18 g/l agar is added.

medium	Ingrediens	pH
NL410 (preculture medium)	10 glucose<br>10 g glycerol<br>5 g oat meal<br>10 g soy flour<br>5 g yeast extract<br>5 g bacto casamino acids<br>1 g $CaCO_3$	pH 7.0
NL19 (main culture medium)	20 g mannitol<br>20 g soy flour	pH 7.5
SGG	10 g soluble starch<br>10 g glucose<br>10 g glycerol<br>2.5 g corn steep powder<br>5 g bacto peptone<br>2 g yeast extract<br>1 g NaCl<br>3 g $CaCO_3$	pH 7.3
MS	20 g mannitol<br>20 g soya flour, full fat	-
R5 medium	103 g saccharose<br>10 g glucose<br>0,25 g K_2SO_4<br>10,12 g $MgCl_2$<br>0,1 g casamino acids<br>5 g yeast extract<br>5,73 g TES<br>2 ml trace element solution<br>in 955 ml $H_2O_{deion.}$ to solve	adjust pH before autoclaving with NaOH to 7.2;<br>after autoclaving separate addition of:<br>20 ml 1 M $CaCl_2$,<br>10 ml 0,54% KH_2PO_4,<br>15 ml 20 % L-proline
Yeast Malt (YM)	10 g malt extract<br>5 g yeast extract<br>4 g glucose	pH 7.3
Oat meal medium (OM)	20 g oat meal<br>5 ml trace element solution	pH 7.3
TSG	17 g bacto tryptone<br>3 g bacto soytone<br>2.5 g glucose<br>5 g NaCl<br>2.5 g K_2HPO_4	set pH to 7.3 before autoclaving

NL300	20 g mannitol<br>20 g cotton seed	pH 7.5
NL333	5 g glucose<br>10 g soluble starch<br>10 g malt extract<br>3 g yeast extract<br>3 g bacto peptone<br>3 g NH_4NO_3<br>2 g $CaCO_3$	pH 7.2
NL500	10 g soluble starch<br>10 g glucose<br>10 g glycerol<br>15 g fish flour<br>10 g sea salt	Adjust pH to 8.0, retitrate to pH 8.0 after 1 hour = pH 6.6 after autoclaving
NL550	10 g soluble starch<br>10 g glucose<br>5 g peptone from casein<br>2.5 g corn starch powder<br>2 g $CaCO_3$<br>10 g sea salt	Adjust pH to 8.0, retitrate to pH 8.0 after 1 hour = pH 6.6 after autoclaving
NL800	5 g glucose<br>10 g glycerol<br>10 g soluble starch<br>5 g soy flour, full fat<br>2 g yeast extract<br>1 g NaCl<br>1 g $CaCO_3$	pH 7.2

Table S3. List of optimal culture conditions (media, time point) and bioactivity profile of nine Indonesian strain isolates.

Strain	Optimal production in liquid medium	Optimal production in solid medium	Optimal incubation time (h) in liquid	Antimicrobial activity against Gram (+)/(-) test bacteria
I6	R5	MS	48	(+), (-)
DHE 7-1	OM	OM	48	(+)
DHE 17-7	SGG	SGG	48	(+)

I3	NL550	MS	72	(+), (-)
14	NL550	MS	72	(+), (-)
I5	MS	MS	96	(+), (-)
SHP 22-7	NL300	NL300	96	(+), (-)
BSE 7-9	NL19	NL19	96	(+), (-)
BSE 7F	NL500	NL500	168	(+), (-)

Table S4. List of predicted BGCs of strain DHE 17-7 derived from antiSMASH analysis. The minus sign (-) indicates the BGC did not have any similarity with any BGCs in the antiSMASH database.

Region	Type	Most similar known region	Similarity
Region 1	NRPS	streptothricin	87%
Region 2	NRPS, T1PKS, NRPS-like, hglE-KS	levorin A3 /C06690 / FR-008-III / candicidin A /UNIIAP5PEF5W7U	95%
Region 3	terpene	lysolipin I	4%
Region 4	indole	5-isoprenylindole-3-carboxylate β-Dglycosyl ester	33%
Region 5	terpene	carotenoid	54%
Region 6	T3PKS	herboxidiene	8%
Region 7	ectoine	ectoine	100%
Region 8	melanin	melanin	60%
Region 9	siderophore	desferrioxamine B/desferrioxamine E	83%
Region 10	NRPS-like	-	-
Region 11	PKS-like, furan	methylenomycin A	9%
Region 12	lanthipeptide	catenulipeptin	60%
Region 13	NRPS	phosphonoglycans	5%
Region 14	terpene	albaflavenone	100%
Region 15	T2PKS	spore pigment	66%
Region 16	siderophore	-	-
Region 17	bacteriocin	-	-
Region 18	terpene	geosmin	100%
Region 19	siderophore	paulomycin	7%
Region 20	NRPS	CDA1b / CDA2a /CDA2b/ CDA3a/ CDA3b / CDA4a /CDA4b	70%
Region 21	NRPS-like	amicetin	79%
Region 22	lanthipeptide	-	-
Region 23	terpene	hopene	100%
Region 24	T1PKS	streptovaricin	31%
Region 25	terpene	versipelostatin	5%
Region 26	bacteriocin	informatipeptin	42%

Region 27	NRPS	coelichelin	100%
Region 28	lanthipeptide	-	-
Region 29	lanthipeptide	SapB	100%
Region 30	T2PKS , butyrolactone	fluostatins M-Q	62%

Table S5. List of predicted BGCs of strain SHP22-7 derived from antiSMASH analysis. The minus sign (-) indicates the BGC did not have any similarity with any BGCs in the antiSMASH database.

Region	Type	Most similar known region	Similarity
Region 1	terpene	albaflavenone	100%
Region 2	T2PKS	spore pigment	66%
Region 3	furan	methylenomycin	9%
Region 4	NRPS-like	granaticin	5%
Region 5	melanin	istamycin	4%
Region 6	ectoine	ectoine	100%
Region 7	NRPS	phosphonoglycans	5%
Region 8	bacteriocin	-	-
Region 9	terpene	geosmin	100%
Region 10	NRPS-like	amicetin	70%
Region 11	NRPS	CDA1a/CDA2a/CDA2b/CDA3a/CDA3b/CDA4a/CDA4b	45%
Region 12	T3PKS	herboxidiene	8%
Region 13	siderophore	desferrioxamine B/desferrioxamine E	100%
Region 14	indole	5-isoprenylindole-3-carboxylate β-D-glycosyl ester	23%
Region 15	terpene	carotenoid	36%
Region 16	T2PKS butyrolactone NRPS	fluostatins	62%
Region 17	terpene	hopene	76%
Region 18	lanthipeptide	-	-
Region 19	T1PKS NRPS	candicidin	90%
Region 20	NRPS arylpolyene	lipopeptide 8D1-1/lipopeptide 8D-2	29%
Region 21	siderophore	grincamycin	8%
Region 22	T1PKS	streptovaricin	26%
Region 23	hglE-KS	sanglifehrin	13%
Region 24	bacteriocin	informatipeptin	57%
Region 25	NRPS	coelichelin	27%

Table S6. List of predicted BGCs of strain I3 derived from antiSMASH analysis. The minus sign (-) indicates the BGC did not have any similarity with any BGCs in the antiSMASH database.

Region	Type	Most similar known region	Similarity
Region 1	siderophore	grincamycin	8%
Region 2	terpene	geosmin	100%
Region 3	bacteriocin	-	-
Region 4	terpene	albaflavenone	100%
Region 5	lassopeptide	aborycin	85%
Region 6	siderophore	desferrioxamine B/desferrioxamine E	83%
Region 7	ectoine	ectoine	100%
Region 8	T2PKS	spore pigment	83%
Region 9	terpene	carotenoid	45%
Region 10	T3PKS	alkylresorcinol	100%
Region 11	NRPS, T1PKS, PKS-like	naphthyridinomycin	100%
Region 12	terpene	hopene	84%
Region 13	PKS-like, T1PKS, hglE-KS	nataxazole	55%
Region 14	bacteriocin	informatipeptin	57%
Region 15	ectoine, NRPS, butyrolactone, other, T1PKS	polyoxypeptin	48%
Region 16	amglyccycl	cetoniacytone A	12%
Region 17	TfuA-related	-	-
Region 18	NRPS-like	rhizomide A/rhizomide B/rhizomide C	100%

Table S7. List of predicted BGCs of strain I4 derived from antiSMASH analysis. The minus sign (-) indicates the BGC did not have any similarity with any BGCs in the antiSMASH database.

Region	Type	Most similar known region	Similarity
Region 1	terpene	hopene	92%
Region 2	siderophore	grincamycin	8%
Region 3	terpene	geosmin	100%
Region 4	bacteriocin	-	-
Region 5	terpene	albaflavenone	100%
Region 6	lassopeptide	aborycin	100%
Region 7	lanthipeptide	venezuelin	100%
Region 8	siderophore	desferrioxamine B/desferrioxamine E	83%
Region 9	T2PKS	spore pigment	83%
Region 10	ectoine	ectoine	100%
Region 11	NRPS	SCO-2138	64%
Region 12	TfuA-related	-	-
Region 13	lanthipeptide	SapB	100%
Region 14	amglyccycl	-	-

Region 15	NRPS, T1PKS, other, butyrolactone, ectoine, lanthipeptide, PKS-like	naphthyridinomycin	100%
Region 16	T3PKS	alkylresorcinol	100%
Region 17	terpene	carotenoid	54%
Region 18	hglE-KS, T1PKS, PKS-like	nataxazole	48%
Region 19	bacteriocin	informatipeptin	57%
Region 20	ectoine	ectoine	100%

Table S8. List of predicted BGCs of strain I5 derived from antiSMASH analysis. The minus sign (-) indicates the BGC did not have any similarity with any BGCs in the antiSMASH database.

Region	Type	Most similar known region	Similarity
Region 1	T3PKS	alkylresorcinol	100%
Region 2	terpene	carotenoid	54%
Region 3	T2PKS	spore pigment	83%
Region 4	ectoine	ectoine	100%
Region 5	NRPS	SCO-2138	64%
Region 6	siderophore	desferrioxamine B/desferrioxamine E	83%
Region 7	lanthipeptide	venezuelin	100%
Region 8	lassopeptide	aborycin	100%
Region 9	terpene	albaflavenone	100%
Region 10	bacteriocin	-	-
Region 11	terpene	geosmin	100%
Region 12	siderophore	grincamycin	11%
Region 13	T2PKS	resistomycin / resistoflavine	88%
Region 14	terpene	hopene	92%
Region 15	bacteriocin	informatipeptin	42%
Region 16	NRPS, ectoine, butyrolactone, other, T1PKS	aurantimycin A	51%
Region 17	NRPS, T1PKS, PKS-like	naphthyridinomycin	57%
Region 18	NRPS	naphthyridinomycin	39%
Region 19	ectoine	ectoine	100%

Table S9. List of predicted BGCs of strain BSE 7F derived from antiSMASH analysis. The minus sign (-) indicates the BGC did not have any similarity with any BGCs in the antiSMASH database

Region	Type	Most similar known region	Similarity
Region 1	ectoine	ectoine	100%
Region 2	NRPS	SCO-2138	64%
Region 3	siderophore	desferrioxamine B/desferrioxamine E	83%
Region 4	lanthipeptide	venezuelin	100%
Region 5	lassopeptide	aborycin	100%
Region 6	phenazine	-	-
Region 7	terpene	albaflavenone	100%
Region 8	bacteriocin	-	-
Region 9	terpene	geosmin	100%
Region 10	siderophore	grincamycin	11%
Region 11	T2PKS	resistomycin /resistoflavine	88%
Region 12	terpene	hopene	92%
Region 13	transAT-PKS	weishanmycin	54%
Region 14	NRPS	weishanmycin	41%
Region 15	bacteriocin	informatipeptin	57%
Region 16	NRPS, ectoine, butyrolactone, other, T1PKS	aurantimycin A	51%
Region 17	terpene, amglyccycl	cetoniacytone A	12%
Region 18	T2PKS	spore pigment	83%
Region 19	terpene	carotenoid	45%
Region 20	T3PKS	alkylresorcinol	100%
Region 21	NRPS, T1PKS	antimycin	25%
Region 22	butyrolactone, ectoine	ectoine	100%
Region 23	NRPS, PKS-like	naphthyridinomycin	100%

Table S10. List of predicted BGCs of strain BSE 7-9 derived from antiSMASH analysis. The minus sign (-) indicates the BGC did not have any similarity with any BGCs in the antiSMASH database

Region	Type	Most similar known region	Similarity
Region 1	butyrolactone, ectoine	ectoine	100%
Region 2	bacteriocin	informatipeptin	57%
Region 3	NRPS	weishanmycin	38%
Region 4	transAT-PKS	weishanmycin	58%
Region 5	terpene	hopene	92%
Region 6	T2PKS	resistomycin/resistoflavine	88%
Region 7	siderophore	grincamycin	11%
Region 8	terpene	geosmin	100%
Region 9	bacteriocin	-	-
Region 10	terpene	albaflavenone	100%
Region 11	phenazine	-	-
Region 12	lassopeptide	aborycin	100%
Region 13	lanthipeptide	venezuelin	100%
Region 14	siderophore	desferrioxamine B/desferrioxamine E	83%
Region 15	NRPS	SCO-2138	64%
Region 16	ectoine	ectoine	100%
Region 17	T2PKS	spore pigment	83%
Region 18	terpene	carotenoid	54%
Region 19	T3PKS	alkylresorcinol	100%
Region 20	NRPS, T1PKS, PKS-like	naphthyridinomycin	100%
Region 21	ectoine, butyrolactone	ectoine	100%
Region 22	NRPS, T1PKS	polyoxypeptin	32%

Table S11. List of predicted BGCs of strain DHE 7-1 derived from antiSMASH analysis. The minus sign (-) indicates the BGC did not have any similarity with any BGCs in the antiSMASH database.

Region	Type	Most similar known region	Similarity
Region 1	lanthipeptide	actinorhodin	9%
Region 2	terpene	pentalenolactone	58%
Region 3	NRPS, lanthipeptide	vioprolide A	33%
Region 4	melanin	melanin	57%
Region 5	T1PKS	foxicins A-D	12%
Region 6	lassopeptide, terpene	isorenieratene	63%
Region 7	T3PKS	herboxidiene	7%
Region 8	PKS-like, butyrolactone	-	-
Region 9	ectoine	ectoine	100%
Region 10	melanin	istamycin	4%

Region 11	siderophore	desferrioxamine	66%
Region 12	T2PKS, bacteriocin	spore pigment	83%
Region 13	NRPS	pepticinnamin E	20%
Region 14	terpene	julichrome Q3-3 /julichrome Q3-5	14%
Region 15	siderophore	-	-
Region 16	transAT-PKS-like, NRPS, transATPKS, bacteriocin	phthoxazolin	20%
Region 17	other, T1PKS, PKS-like	meilingmycin	6%
Region 18	bacteriocin	-	-
Region 19	terpene	geosmin	100%
Region 20	siderophore	-	-
Region 21	NRPS, T1PKS	polyoxypeptin	21%
Region 22	terpene	hopene	84%
Region 23	NRPS, T1PKS	conglobatin	26%
Region 24	NRPS, bacteriocin	surugamide A/ surugamide D	19%
Region 25	NRPS	stenothricin	18%
Region 26	other, T3PKS	A-503083 A / A-503083 B / A-503083 E / A-503083 F	9%
Region 27	NRPS	lipopeptide 8D1-1 / lipopeptide 8D1-2	13%

Table S12. List of predicted BGCs of strain I6 derived from antiSMASH analysis. The minus sign (-) indicates the BGC did not have any similarity with any BGCs in the antiSMASH database.

Region	Type	Most similar known region	Similarity
Region 1	indole	staurosporine	100%
Region 2	NRPS	scabichelin	100%
Region 3	NRPS-like, terpene	stenothricin	13%
Region 4	T2PKS, siderophore	spore pigment	75%
Region 5	siderophore	desferrioxamine B/desferrioxamine E	83%
Region 6	thiopeptide, LAP	-	-
Region 7	phosphonate, NRPS	phosphonoacetic	15%
Region 8	NRPS-like	phosphonoglycans	3%
Region 9	NRPS-like	stenothricin	13%
Region 10	siderophore	ficellomycin	3%
Region 11	NRPS	echinomycin	88%
Region 12	bacteriocin	-	-
Region 13	T3PKS	flaviolin	75%
Region 14	terpene	isorenieratene	100%
Region 15	linaridin	nogalamycin	30%
Region 16	bacteriocin	-	-
Region 17	terpene	hopene	61%
Region 18	NRPS, T1PKS	tirandamycin	100%
Region 19	melanin	melanin	28%
Region 20	thiopeptide, LAP	-	-

Region 21	T1PKS, hglE-KS, lanthipeptide	SapB	100%
Region 22	T3PKS, fused	pheganomycin	52%
Region 23	NRPS-like	streptonigrin	5%
Region 24	NRPS, T1PKS, butyrolactone	neocarzinostatin	50%

Table S13. Parameters used in MetaboScape analysis.

Parameter name	Value
Intensity threshold [counts]	1000.0
Minimum peak length [spectra]	3
Minimum peak length (recursive) [spectra]	1
Minimum # Features for Extraction	1
Presence of features in minimum # of analyses	1
Lock mass calibration	false
Mass calibration	false
Excl. mass list	[666.019887, 413.0, 352.0]
Excl. mass tolerance	5.0
Excl. mass tolerance unit	mDa
Primary Ion	$[M+H]^+$
Seed Ions	$[M+Na]^+$, $[M+K]^+$, $[2M+H]^+$, $[2M+Na]^+$, $[M+2H]^{2+}$, $[M+H+Na]^{2+}$
Common Ions	$[M-H_2O+H]^+$, $[M+H_2O+H]^+$, $[2M+H_2O+H]^+$, $[2M-H_2O+H]^+$
EIC correlation	0.8
Mass range: Start [m/z]	150.0
Mass range: End [m/z]	1800.0
Retention time range: Start [min]	1.0
Retention time range: End [min]	48.0
Perform MS/MS import	true
Group by collision energy	true
MS/MS import method	average

References:

1. Luo, X.X.; Kai, L.; Wang, Y.; Wan, C.X.; Zhang, L.L. *Streptomyces luteus* sp. nov., an actinomycete isolated from soil. *Int. J. Syst. Evol. Microbiol.* **2017**, *67*, 543–547, doi:10.1099/ijsem.0.001611.
2. Mertz, F.P.; Higgens, C.E. *Streptomyces capillispiralis* sp. nov. *Int. J. Syst. Bacteriol.* **1982**, *32*, 116–124, doi:10.1099/00207713-32-1-116.
3. Eguchi, T.; Takada, N.; Nakamura, S.; Tanaka, T.; Makino, T.; Oshima, Y. *Streptomyces bungoensis* sp. nov. *Int. J. Syst. Bacteriol.* **1993**, doi:10.1099/00207713-43-4-794.
4. Huang, X.; Zhou, S.; Huang, D.; Chen, J.; Zhu, W. *Streptomyces spongiicola* sp. Nov., An actinomycete derived from marine sponge. *Int. J. Syst. Evol. Microbiol.* **2016**, *66*, 738–743, doi:10.1099/ijsem.0.000782.
